# A comparison of analytic approaches for individual patient data meta-analyses with binary outcomes

**DOI:** 10.1186/s12874-017-0307-7

**Published:** 2017-02-16

**Authors:** Doneal Thomas, Robert Platt, Andrea Benedetti

**Affiliations:** 1 Department of Epidemiology, Biostatistics & Occupational Health, McGill University, Montreal, Canada; 2 Department of Medicine, McGill University, Montreal, Canada; 3 Respiratory Epidemiology and Clinical Research Unit, McGill University Health Centre, Purvis Hall, 1020 Pine Avenue West, Montreal, QC H3A 1A2 Canada

**Keywords:** Individual patient data meta-analyses, One- and two-stage models, Generalized linear mixed models, Penalized quasi-likelihood, Adaptive gauss-hermite quadrature, Fixed and random study-effects

## Abstract

**Background:**

Individual patient data meta-analyses (IPD-MA) are often performed using a one-stage approach-- a form of generalized linear mixed model (GLMM) for binary outcomes. We compare (i) one-stage to two-stage approaches (ii) the performance of two estimation procedures (Penalized Quasi-likelihood-PQL and Adaptive Gaussian Hermite Quadrature-AGHQ) for GLMMs with binary outcomes within the one-stage approach and (iii) using stratified study-effect or random study-effects.

**Methods:**

We compare the different approaches via a simulation study, in terms of bias, mean-squared error (MSE), coverage and numerical convergence, of the pooled treatment effect (*β*_1_) and between-study heterogeneity of the treatment effect (*τ*_1_^2^). We varied the prevalence of the outcome, sample size, number of studies and variances and correlation of the random effects.

**Results:**

The two-stage and one-stage methods produced approximately unbiased *β*_1_ estimates. PQL performed better than AGHQ for estimating *τ*_1_^2^ with respect to MSE, but performed comparably with AGHQ in estimating the bias of *β*_1_ and of *τ*_1_^2^. The random study-effects model outperformed the stratified study-effects model in small size MA.

**Conclusion:**

The one-stage approach is recommended over the two-stage method for small size MA. There was no meaningful difference between the PQL and AGHQ procedures. Though the random-intercept and stratified-intercept approaches can suffer from their underlining assumptions, fitting GLMM with a random-intercept are less prone to misfit and has good convergence rate.

**Electronic supplementary material:**

The online version of this article (doi:10.1186/s12874-017-0307-7) contains supplementary material, which is available to authorized users.

## Background

Individual Patient Data (IPD) meta-analyses (MA) are regarded as the gold standard in evidence synthesis and are increasingly being used in current practice [1, 2]. However, the implementation of the analysis of IPD-MA requires additional expertise and choices [3], particularly when the outcome is binary. These include (i) should a one- or two-stage model be used [4, 5], (ii) what estimation procedure should be used to estimate the one-stage model [6, 7] and, (iii) should the study effect be fixed or random [8].

Although IPD-MA were conventionally analyzed via a two-stage approach [9], over the last decade, use of the one-stage approach has increased [10]. Recently, some have suggested that the two-stage and one-stage frameworks produce similar results for MA of large randomized controlled trials [5]. The literature suggests the one-stage method is particularly preferable when few studies or few events are available as it uses a more exact statistical approach than relying on a normality approximation [3–5].

When IPD are available and the outcome is binary, the one-stage approach consists of estimating Generalized Linear Mixed Models (GLMMs) with a random slope for the exposure, to allow the exposure effect to vary across studies. Penalized quasi-likelihood (PQL) introduced by Breslow and Clayton is a popular method for estimating the parameters in GLMMs [11]. However, regression parameters can be badly biased for some GLMMs, especially with binary outcomes with few observations per cluster, low outcome rates, or high between cluster variability [12, 13]. Adaptive Gaussian Hermite quadrature (AGHQ) is the current favored competitor to PQL, which approximates the maximum likelihood by numerical integration [14]. Although estimation becomes more precise as the number of quadrature points increases, it often gives rise to computational difficulties for high-dimension random effects and convergence problems where variances are close to zero or cluster sizes are small [14].

The heterogeneity between studies is an important aspect to consider when carrying out IPD-MA. Such heterogeneity may arise due to differences in study design, treatment protocols or patient populations [8]. When such heterogeneity is present, the convention is to include a random slope in the model as it captures the variability of the exposure across studies. However, there are corresponding assumptions in regards to the study effect being modelled as stratified or random [4, 15].

Few comparisons of GLMMs have been reported in the context of IPD-MA with binary outcomes [4, 15], that is, when the number of studies and the number of subjects within each study is small, study sizes are imbalanced, in the presence of large between-study heterogeneity and small exposure effects and there is an interest in the variance parameter of the random treatment effect. According to previous literature, these factors have all been identified as influencing model performance [6]. While several simulation studies have been published, these have mainly limited their attention to simple models with only random intercepts [13, 16]. Thus, the performance of the random effects models including both a random intercept and a random slope are less well known.

Our objective was to assess and compare via simulation studies, (i) one-stage approaches to conventional two-stage approaches (ii) the performance of different estimation procedures for GLMMs with binary outcomes, and (iii) using stratified study-effect or random study-effects in a randomized trial setting. We use our results to develop guidelines on the choice of methods for analyzing data from IPD-MA with binary outcomes and to understand explicitly the trade-offs between computational and statistical complexity.

Methods section introduces the models we are considering, the design of the simulation study and the assessment criteria. In Results section, results for the different methods under varying conditions are presented and discussed. Discussion section concludes with a discussion.

## Methods

We conducted a simulation study to compare various analytic approaches to analyze data from IPD-MA with binary outcomes. Hereto, our methods all assume that between-study heterogeneity exists, as it is likely in practice, and so only random treatment-effects IPD meta-analysis models are considered.

### Data Generation

The data generation algorithm was developed to generate two-level data sets (e.g. patients grouped into studies). We generated a binary outcome (*Y*_*ij*_) and a single binary exposure (*X*_*ij*_). We denote the number of studies *j* = 1, 2 …, *K* and *i* = 1, 2 …, *n*_*j*_ denotes the individuals per study. Therefore, *Y*_*ij*_ is the outcome observed for the *i* ^*th*^ individual from the *j* ^*th*^ study.

The dichotomous exposure variable, *X*_*ij*_, was generated from a Bernoulli distribution with probability = 0.5 and recoded $$ \pm {\scriptscriptstyle \raisebox{1ex}{$1$}\!\left/ \!\raisebox{-1ex}{$2$}\right.} $$ to indicate control/treatment group [15]. To generate the binary outcome variable *Y*_*ij*_, first the probability of the outcome was calculated from the random-study and –treatment effects logistic regression model (Eq. 1), or the stratified-study effects model (Eq. 2):

1 $$ logit\left({\pi}_{ij}\right)=\left({\beta}_0+{b}_{0 j}\right)+\left({\beta}_1+{b}_{1 j}\right){x}_{ij} $$

2 $$ logit\left({\pi}_{ij}\right)={\beta}_j+\left({\beta}_1+{b}_{1 j}\right){x}_{ij} $$

Here *π*_*ij*_ is the true probability of the outcome for the *i* ^*th*^ individual from the *j* ^*th*^ study, *β*_0_denotes the mean log-odds of the outcome (study-effect) and *β*_1_ the pooled treatment effect (log odds ratio). The random effects (*b*_0*j*_ and *b*_1*j*_) were generated from a bivariate normal distribution with mean = 0 and variance-covariance matrix $$ \Sigma =\left(\begin{array}{cc}\hfill {\sigma}^2\hfill & \hfill \rho \sigma \tau \hfill \\ {}\hfill \rho \sigma \tau \hfill & \hfill {\tau}^2\hfill \end{array}\right) $$ for the random study-effect case. In the stratified study effects case, (i.e. Eq. (2)), *β*_*j*_, were generated from a uniform distribution and *b*_1*j*_ was generated from a normal distribution with zero mean and variance, *τ* ^2^.

A Bernoulli distribution with probability *π*_*ij*_ from Eqs. (1) and (2) was used to generate the binary outcome *Y*_*ij*_.

The number of studies, study size, total sample size, variances and correlation of the random effects, and average conditional probability were all varied, with levels described in Table 1. For each distinct combination (*n* = 480) of simulation parameters, 1000 IPD-MA were generated from each Eqs. (1) and (2), allowing us to investigate a wide range of scenarios. The heterogeneity was set at I^2^ = 0.01, 0.23 and 0.55 as defined by *τ* ^2^/(*τ* ^2^ + *π* ^2^/3) for a binary outcome using an odds ratio [17]. The levels correspond to: little or no, low and moderate heterogeneity respectively [18].

**Table 1:** Summary of Simulation Parameters^a^

Parameters	Values
IPD-Meta-analyses generated:	M = 1000
(Number of studies, number of subjects per study, total average sample sizes)^b^:	(*K*, *n*_*i*_, *N*) ∈ {(5,100,500), (15,33,500), (15,200,3000), **(5,357,500)**, **(15,98,500)**, **(15,588,3000)**}
Fixed effects (intercepts):	*β*_0_ = − 0.85
Prevalence of the outcome	*π* = 30%
Fixed effects (Slopes):	*β*_1_ = 0.18
Random effects distribution:	Normal
Random effects variances:	{*τ*_0_^2^, *τ*_1_^2^} ∈ (0.05, 1, 4)
Correlation between random effects:	*ρ* ∈ (0,0.5)

^a^In a sensitivity analysis, we extended the number of studies to 50 with an average sample size of 9000 and reduced the prevalence of the outcome to 5%. The prevalence of the outcome was fixed to 30% by setting the value of the intercept *β*_0_ to –0.85

^b^The number of subjects per study was reported for only large studies when data sets were generated with imbalanced study sizes (bold text: 25% large studies-10 times more subjects)

A sensitivity analysis was also considered to explore the performance of different methods when just 5% of observation had a positive outcome.

### Models

#### Two-stage IPD methods

In the two-stage approach, each study in the IPD was analyzed separately via logistic regression

$$ {y}_i\sim Bernoulli\left({p}_i\right) $$

$$ logit\left({p}_{i j}\right)={\gamma}_0+{\gamma}_1{x}_i $$

The first step estimated the study-specific intercept and slope and their associated within-study covariance matrix (consisting of the variances of the intercept and slope, as well as the covariance) for each study. This model reduces the IPD to its relative treatment effect estimate and variance for each study then at the second stage these aggregate data (AD) are synthesized (described below).

##### Model 1- Bivariate meta-analysis

The AD were combined via a bivariate random-effects model that simultaneously synthesized the estimates whilst accounting for their correlation, and the within-study correlation [4]. The model assumes that the true effects follow a bivariate normal distribution and is estimated via restricted maximum likelihood with the following marginal distributions of the estimates [19]:

$$ \left[\begin{array}{c}\hfill \widehat{\gamma_{0 J}}\hfill \\ {}\hfill \widehat{\gamma_{1 J}}\hfill \end{array}\right]\sim N\left(\left(\begin{array}{c}\hfill {\gamma}_0\hfill \\ {}\hfill {\gamma}_1\hfill \end{array}\right),\varSigma +{C}_j\right),\varSigma =\left(\begin{array}{c}\hfill {\tau}_0^2\hfill \\ {}\hfill {\tau}_{01}^2\hfill \end{array}\begin{array}{c}\hfill {\tau}_{01}^2\hfill \\ {}\hfill {\tau}_1^2\hfill \end{array}\right) $$

where Σ is the unknown between-study variance-covariance matrix of the true effects (*γ*_0_ *and γ*_1_) and *C*_*j*_ (*j* = 1, …, *K*) the with-in study variance-covariance matrix with the variances of the estimates.

##### Model 2: Conventional DerSimonian and Laird approach

The with-in study and between-study covariance estimates are often times not estimated since most researchers assumed that studies are independent, and instead a univariate meta-analysis of the logit of the odds ratios is performed [20]. The marginal distribution of the pooled estimated treatment effect under this approach is easily obtained as:

$$ \widehat{\gamma_{1 J}}\sim N\left({\gamma}_1,{\tau}_1^2+ var\left(\widehat{\gamma_J}\right)\right) $$

with unknown parameters *γ*_1_ and *τ*_1_^2^, estimated via the inverse variance weighted non-iterative method (method-of-moments) [21].

#### One-stage IPD methods

The one-stage approach analyzes the IPD from all studies simultaneously, while accounting for clustering of subjects within studies [4]. The one-stage model is a form of GLMM. Two different specifications are considered.

##### Model 3- Random intercept and random slope

We estimated a GLMM with a random study effect *u*_0*j*_ and a random treatment effect *u*_1*j*_ via PQL and AGHQ, and allowed the random effects to be correlated, which implies that the between-study covariance between *u*_0*j*_ and *u*_1*j*_ is fully estimated.

$$ \begin{array}{l} logit\left({p}_{ij}\right) = {\gamma}_0+{u}_{0 j}+\left({\gamma}_1+{u}_{1 j}\right){x}_{ij}\\ {}\left[\begin{array}{c}\hfill {u}_{0 j}\hfill \\ {}\hfill {u}_{1 j}\hfill \end{array}\right]\sim N\left(\left(\begin{array}{c}\hfill 0\hfill \\ {}\hfill 0\hfill \end{array}\right),{\Sigma}_j\right),\ {\Sigma}_j=\left(\begin{array}{cc}\hfill {\tau}_0^2\hfill & \hfill {\tau}_{01}^2\hfill \\ {}\hfill {\tau}_{01}^2\hfill & \hfill {\tau}_1^2\hfill \end{array}\right)\end{array} $$

##### Model 4-Stratified intercept one-stage

Finally, the stratified one-stage approach estimates a separate intercept for each study rather than constraining the intercepts to follow a normal or other distribution. Therefore, there is no need for the normality assumption for the study membership, hence, the between-study covariance term is no longer estimated. The model is defined as follows:

$$ logit\left({p}_{ij}\right) = {\displaystyle \sum_{k=1}^K}\left({\gamma}_k{I}_{k= j}\right)+\left({\gamma}_1+{u}_{1 j}\right){x}_{ij} $$

where *I*_*k* = *j*_ indicates that a separate intercept should be estimated for each study *j* = 1, …, *K* and *u*_1*j*_ ~ *N*(0, *τ*_1_^2^). Parameters of both Models 3 and 4 were estimated via PQL and AGHQ.

### Estimation Procedures and Approximations

The parameters of the one-stage models were estimated using PQL and AGHQ. For the two-stage approach, a logistic regression was first estimated for each study via maximum likelihood. The parameters of the two-stage model were estimated via method-of-moments (MOM) (Model 2) and restricted maximum likelihood (REML) (Model 1) [21–23] at the second stage.

Both likelihood-based methods (PQL and AGHQ) were implemented on SAS version 9.4 using PROC GLIMMIX with default options [24]. The number of quadrature points in AGHQ was selected automatically [25], the absolute value for parameter convergence criterion was 10–8 and the maximum number of iterations was 100.

Therefore, for each generated data set the following models were fit.

- Two-stage approach (Models 1 and 2)
- One-stage approach via GLMMs (Models 3 and 4) estimated with PQL.
- One-stage approach via GLMMs (Models 3 and 4) estimated with AGHQ.

#### Assessment criteria

The performance of the estimation methods was evaluated using: a) numerical convergence, b) absolute bias; c) root mean square error (RMSE); and d) coverage probability - of the pooled treatment effect and its between-study variability.

##### Numerical convergence

The convergence rate was estimated for all models fit, as the number of simulation repetitions that did converge (without returning a warning message) divided by the total attempted (M = 1000). Models that returned a warning message specifying that the estimated variance-covariance matrix was not positive definite or that the optimality condition was violated were considered not to have converged.

##### Bias

The Monte Carlo bias of the pooled treatment effect and its between-study heterogeneity is defined as the average of the bias in the estimates provided by each method as compared to the truth, across the 1000 IPD-MA in each scenario. The Monte Carlo estimate of the bias is computed as

$$ bias=\frac{1}{1000}{\displaystyle \sum_{j=1}^{1000}}\ {\widehat{\theta}}_j-\theta, $$

where ${\hat{\theta}}_j$ were the parameter estimates and *θ* was the true parameter of the pooled treatment effect or its between-study variance. We also reported the mean absolute bias (AB).

##### Mean square error

The mean square error (MSE) is a useful measure of the overall accuracy, because it penalizes an estimate for both bias and inefficiency. The Monte Carlo estimate of the MSE is:

$$ M S E\left(\widehat{\theta}\right)=\frac{1}{1000}{\displaystyle \sum_{j=1}^{1000}}{\widehat{\Big(\theta}}_j-\theta \Big){}^2, $$

For each scenario, the RMSE of the pooled treatment effect and its between-study heterogeneity was reported, as this measure is on the same scale as the parameter.

##### Coverage probability

We estimated coverage for the pooled treatment effect and its between-study heterogeneity for the various methods. Gaussian coverage was estimated, where if $\left|\hat{\theta}-\theta \right|\le 1.96\times S E\left(\hat{\theta}\right)$ the true value was covered, and if $\left|\hat{\theta}-\theta \right|>1.96\times S E\left(\hat{\theta}\right)$ it was not.

We reported the median, the 25^th^ and 75^th^ percentiles of the AB and RMSE of the pooled treatment effect and its between-study heterogeneity but reported percentages for the numerical convergence and coverage rate.

## Results

Tables 2, 3, 4, 5, 6 and 7 present the median and interquartile range of the AB, RMSE, coverage and convergence of the pooled treatment effect and its between-study variance, respectively, as estimated via two- and one-stage; AGHQ and PQL; random-intercept and stratified-intercept methods. We reported results for data generated with imbalances in study sizes (different sample size in all studies) for both the random-intercept and stratified-intercept data generation (Eqs. 1 and 2) with correlated random effects (*ρ* = 0.5), as this scenario is likely the closest to real-life.

**Table 2:** Performance of the one- and two-stage approaches in small data sets^a^ with greater (Top panel) and lesser (Bottom panel) heterogeneity of random effects^b^

		Data generation			
	Performance measures^c^	Random-study and treatment effect (Eq. 1)		Stratified-study effect (Eq. 2)	
		Two-stage^d^	One-stage	Two-stage	One-stage
(*τ*_0_^2^, *τ*_1_^2^) = (4, 4) ^e^	AB (*β*_1_)	0.04 (0.02 0.06)	0.04 (0.02, 0.07)	0.04 (0.02, 0.06)	0.04 (0.01, 0.07)
	RMSE (*β*_1_)	**1.11 (0.49, 1.94)**	1.19 (0.53, 2.12)	**1.18 (0.59, 1.96)**	1.23 (0.61, 2.14)
	Coverage (*β*_1_)	89.3	**91.8**	92	**92.6**
	AB (*τ*_1_^2^)	0.23 (0.14,0.30)	**0.16 (0.08, 0.24)**	**0.15 (0.08,0.24)**	0.24 (0.20, 0.27)
	RMSE (*τ*_1_^2^)	7.26 (4.39,7.51)	**4.93 (2.56, 7.51)**	**4.81 (2.38,7.42)**	7.47 (6.28, 8.64)
	Coverage (*τ*_1_^2^) ^f^	NA	NA	NA	NA
	Convergence	**100**	97.7	**100**	99.8
(*τ*_0_^2^, *τ*_1_^2^) = (1, 1)	AB (*β*_1_)	0.02 (0.01, 0.04)	0.02 (0.01, 0.04)	0.03 (0.01, 0.04)	0.03 (0.01, 0.04)
	RMSE (*β*_1_)	**0.73 (0.35, 1.29)**	0.75 (0.37, 1.33)	0.80 (0.37, 1.30)	**0.79 (0.39, 1.34)**
	Coverage (*β*_1_)	89.1	**90.6**	91.1	**91.6**
	AB (*τ*_1_^2^)	0.06 (0.03, 0.08)	**0.04 (0.02, 0.1)**	0.05 (0.03, 0.08)	**0.03 (0.01, 0.07)**
	RMSE (*τ*_1_^2^)	1.73 (0.85, 2.65)	**1.22 (0.53, 3.16)**	1.59 (0.80, 2.46)	**1.06 (0.46, 2.06)**
	Coverage (*τ*_1_^2^)	NA	NA	NA	NA
	Convergence	**100**	90.4	100	100

^a^Small data sets had 15 studies and on average 500 total subjects

^b^Bold text represent “best value” of performance

^c^Median (25th and 75th percentile) were reported for AB and RMSE, the proportion was reported for coverage and convergence

^d^Two-stage method via conventional DerSimonian and Laird (Model 2). One-stage (Random-intercept and random treatment effect with PQL (Model 3)

^e^(*τ*_0_^2^, *τ*_1_^2^): (Random treatment-effect variance, random study-effect variance)

^f^The two-stage approach did not return a confidence interval for *τ*_1_^2^, hence no coverage was estimated and comparison was not applicable (NA) to the one-stage method

**Table 3:** Performance of the one- and two-stage approaches in large data sets^a^ with greater (Top panel) and lesser (Bottom panel) heterogeneity of random effects^b^

		Data generation			
	Performance measures^c^	Random-study and treatment effect (Eq. 1)		Stratified-study effect (Eq. 2)	
		Two-stage^d^	One-stage	Two-stage	One-stage
(*τ*_0_^2^, *τ*_1_^2^) = (4, 4) ^e^	AB (*β*_1_)	0.03 (0.02 0.06)	0.03 (0.02, 0.06)	0.04 (0.01, 0.06)	0.04 (0.01, 0.06)
	RMSE (*β*_1_)	**1.02 (0.50, 1.85)**	1.07 (0.49, 1.84)	1.15 (0.57, 1.93)	**1.11 (0.58, 1.87)**
	Coverage (*β*_1_)	91.9	**92.3**	92.4	**93.6**
	AB (*τ*_1_^2^)	0.14 (0.07,0.22)	**0.12 (0.06, 0.20)**	**0.11 (0.05,0.17)**	0.22 (0.20, 0.25)
	RMSE (*τ*_1_^2^)	4.36 (2.22,6.80)	**3.87 (1.81, 6.21)**	**3.40 (1.70,5.47)**	6.99 (6.20, 7.80)
	Coverage (*τ*_1_^2^) ^f^	NA	NA	NA	NA
	Convergence	**100**	98.3	**100**	89.9
(*τ*_0_^2^, *τ*_1_^2^) = (1, 1)	AB (*β*_1_)	0.02 (0.01, 0.03)	0.02 (0.01, 0.03)	0.02 (0.01, 0.03)	0.02 (0.01, 0.03)
	RMSE (*β*_1_)	0.61 (0.30, 1.04)	**0.59 (0.29, 1.05)**	**0.61 (0.30, 1.02)**	0.63 (0.30, 1.03)
	Coverage (*β*_1_)	91.2	**91.9**	93	**93.3**
	AB (*τ*_1_^2^)	0.03 (0.02, 0.06)	0.03 (0.02, 0.05)	0.03 (0.02, 0.05)	**0.02 (0.01, 0.03)**
	RMSE (*τ*_1_^2^)	1.08 (0.53, 1.73)	**1.03 (0.49, 1.68)**	1.00 (0.51, 1.68)	**0.57 (0.27, 1.00)**
	Coverage (*τ*_1_^2^)	NA	NA	NA	NA
	Convergence	**100**	96.5	**100**	88.8

^a^Large data sets had 15 studies and on average 3000 total subjects

^b^Bold text represent “best value” of performance

^c^Median (25th and 75th percentile) were reported for AB and RMSE, the proportion was reported for coverage and convergence

^d^Two-stage method via conventional DerSimonian and Laird (Model 2). One-stage (Random-intercept and random treatment effect with PQL (Model 3)

^e^(*τ*_0_^2^, *τ*_1_^2^): (Random treatment-effect variance, random study-effect variance)

^f^The two-stage approach did not return a confidence interval for *τ*_1_^2^, hence no coverage was estimated and comparison was not applicable (NA) to the one-stage method

**Table 4:** Performance of Penalized Quasi-likelihood and Adaptive Gaussian Hermite Quadrature estimation approaches in small data sets^a^ with greater (Top panel) and lesser (Bottom panel) heterogeneity of random effects^b^

	Performance measures^c^	Data generation			
		Random-study and treatment effect (Eq. 1)		Stratified-study effect (Eq. 2)	
		AGHQ^d^	PQL^d^	AGHQ	PQL
(*τ*_0_^2^, *τ*_1_^2^) = (4, 4)^e^	AB ($\beta$_1_)	0.05 (0.02, 0.08)	**0.04 (0.02, 0.07)**	0.04 (0.02, 0.07)	0.04 (0.02, 0.07)
	RMSE ($\beta$_1_)	1.42 (0.64, 2.52)	**1.19 (0.53, 2.12)**	1.35 (0.65, 2.27)	**1.23 (0.61, 2.14)**
	Coverage ($\beta$_1_)	**93.2**	91.8	91.7	**92.6**
	AB (*τ*_1_^2^)	0.18 (0.09,0.29)	**0.16 (0.08, 0.24)**	**0.15 (0.08,0.24)**	0.24 (0.20, 0.27)
	RMSE (*τ*_1_^2^)	5.76 (2.80,9.07)	**4.93 (2.56, 7.51)**	**4.79 (2.37,7.62)**	7.47 (6.28, 8.64)
	Coverage (*τ*_1_^2^)	**85.5**	76.2	**81.4**	4.6
	Convergence	**99**	97.7	96.7	**99.8**
(*τ*_0_^2^, *τ*_1_^2^) = (1, 1)	AB ($\beta$_1_)	0.03 (0.01, 0.05)	**0.02 (0.01, 0.04)**	0.03 (0.01, 0.04)	0.03 (0.01, 0.04)
	RMSE ($\beta$_1_)	0.79 (0.41, 1.42)	**0.75 (0.37, 1.33)**	0.84 (0.42, 1.38)	**0.79 (0.39, 1.34)**
	Coverage ($\beta$_1_)	**92.3**	90.6	**93.4**	91.6
	AB (*τ*_1_^2^)	0.06 (0.03, 0.09)	**0.04 (0.02, 0.1)**	0.05 (0.02, 0.08)	**0.03 (0.02, 0.07)**
	RMSE (*τ*_1_^2^)	1.76 (0.84, 2.70)	**1.22 (0.53, 3.16)**	1.54 (0.72, 2.40)	**1.06 (0.46, 2.06)**
	Coverage (*τ*_1_^2^)	74.5	**81.1**	71.6	**77.2**
	Convergence	**96.8**	90.4	85.8	**100**

^a^Small data sets had 15 studies and on average 500 total subjects

^b^Bold text represent “best value” of performance

^c^Median (25th and 75th percentile) were reported for AB and RMSE, the proportion was reported for coverage and convergence

^d^Results are given for Adaptive Gaussian Hermite Quadrature (AGHQ) and Penalized Quasi-likelihood (PQL) for the One-stage random-intercept and random treatment effect model (Model 3)

^e^(*τ*_0_^2^, *τ*_1_^2^): (Random treatment-effect variance, random study-effect variance)

**Table 5:** Performance of Penalized Quasi-likelihood and Adaptive Gaussian Hermite Quadrature estimation approaches in large data sets^a^ with greater (Top panel) and lesser (Bottom panel) heterogeneity of random effects^b^

	Performance measures^c^	Data generation			
		Random-study and treatment effect (Eq. 1)		Stratified-study effect (Eq. 2)	
		AGHQ^d^	PQL^d^	AGHQ	PQL
(*τ*_0_^2^, *τ*_1_^2^) = (4, 4)^e^	AB ($\beta$_1_)	0.04 (0.02, 0.06)	**0.03 (0.02, 0.06)**	0.04 (0.01, 0.06)	0.04 (0.01, 0.06)
	RMSE ($\beta$_1_)	1.20 (0.55, 1.99)	**1.07 (0.49, 1.84)**	1.16 (0.58, 1.95)	**1.11 (0.58, 1.87)**
	Coverage ($\beta$_1_)	92.2	**92.3**	92.1	**93.6**
	AB (*τ*_1_^2^)	0.13 (0.07,0.21)	**0.12 (0.06, 0.20)**	**0.11 (0.05,0.18)**	0.22 (0.20, 0.25)
	RMSE (*τ*_1_^2^)	4.12 (2.06,6.77)	**3.87 (1.81, 6.21)**	**3.42 (1.71,5.77)**	6.99 (6.20, 7.80)
	Coverage (*τ*_1_^2^)	**81.9**	78.9	**84.7**	1.0
	Convergence	**100**	98.3	**100**	89.9
(*τ*_0_^2^, *τ*_1_^2^) = (1, 1)	AB ($\beta$_1_)	0.02 (0.01, 0.03)	0.02 (0.01, 0.03)	0.02 (0.01, 0.03)	0.02 (0.01, 0.03)
	RMSE ($\beta$_1_)	0.60 (0.30, 1.06)	**0.59 (0.29, 1.05)**	0.63 (0.30, 1.05)	0.63 (0.30, 1.03)
	Coverage ($\beta$_1_)	91.7	**91.9**	92.4	**93.3**
	AB (*τ*_1_^2^)	0.04 (0.02, 0.06)	**0.03 (0.02, 0.05)**	0.03 (0.02, 0.05)	**0.02 (0.01, 0.03)**
	RMSE (*τ*_1_^2^)	1.09 (0.52, 1.75)	**1.03 (0.49, 1.68)**	1.01 (0.49, 1.69)	**0.57 (0.27, 1.00)**
	Coverage (*τ*_1_^2^)	**83.6**	82.5	**82.5**	76.5
	Convergence	**99.5**	96.5	**99.1**	88.8

^a^Large data sets had 15 studies and on average 3000 total subjects

^b^Bold text represent “best value” of performance

^c^Median (25th and 75th percentile) were reported for AB and RMSE, the proportion was reported for coverage and convergence

^d^Results are given for Adaptive Gaussian Hermite Quadrature (AGHQ) and Penalized Quasi-likelihood (PQL) for the One-stage random-intercept and random treatment effect model (Model 3)

^e^(*τ*_0_^2^, *τ*_1_^2^): (Random treatment-effect variance, random study-effect variance)

**Table 6:** Performance of the stratified- and random-intercept^a^ models approaches in small data sets^b^ with greater (Top panel) and lesser (Bottom panel) heterogeneity of random effects^c^

	Performance measures^d^	Data generation			
		Random-study and -treatment effect (Eq. 1)		Stratified-study effect (Eq. 2)	
		Stratified-intercept	Random-intercept	Stratified-intercept	Random-intercept
(*τ*_0_^2^, *τ*_1_^2^) = (4, 4)^e^	AB ($\beta$_1_)	0.04 (0.02, 0.08)	0.04 (0.02, 0.07)	0.05 (0.02, 0.07)	**0.04 (0.01, 0.07)**
	RMSE ($\beta$_1_)	1.24 (0.49, 2.44)	**1.19 (0.53, 2.12)**	1.43 (0.70, 2.32)	**1.23 (0.61, 2.14)**
	Coverage ($\beta$_1_)	**99.1**	91.8	**97.4**	92.6
	AB (*τ*_1_^2^)	0.16 (0.07, 0.25)	0.16 (0.08, 0.24)	**0.15 (0.07,0.24)**	0.24 (0.20, 0.27)
	RMSE (*τ*_1_^2^)	5.01 (2.35,7.95)	**4.93 (2.56, 7.51)**	**4..75 (2.23,7.64)**	7.47 (6.28, 8.64)
	Coverage (*τ*_1_^2^)	11.6	**76.2**	**28.4**	4.6
	Convergence	13.8	**97.7**	32.3	**99.8**
(*τ*_0_^2^, *τ*_1_^2^) = (1, 1)	AB ($\beta$_1_)	0.03 (0.01, 0.04)	**0.02 (0.01, 0.04)**	0.03 (0.01, 0.05)	0.03 (0.01, 0.04)
	RMSE ($\beta$_1_)	0.83 (0.41, 1.38)	**0.75 (0.37, 1.33)**	0.90 (0.42, 1.47)	**0.79 (0.39, 1.34)**
	Coverage ($\beta$_1_)	**96.4**	90.6	**94**	91.6
	AB (*τ*_1_^2^)	0.05 (0.03, 0.09)	**0.04 (0.02, 0.1)**	0.05 (0.02, 0.08)	**0.03 (0.01, 0.07)**
	RMSE (*τ*_1_^2^)	1.72 (0.85, 2.78)	**1.22 (0.53, 3.16)**	1.55 (0.75, 1.61)	**1.06 (0.46, 2.06)**
	Coverage (*τ*_1_^2^)	37.3	**81.1**	54.4	**77.2**
	Convergence	42.6	**90.4**	62.3	**100**

^a^Results are given for Penalized Quasi-likelihood (PQL) for the One-stage random-intercept and random treatment effect model (Model 3) and the stratified-intercept and random-slope model (Model 4)

^b^Small data sets had 15 studies and on average 500 total subjects

^c^Bold text represent “best value” of performance

^d^Median (25th and 75th percentile) were reported for AB and RMSE, the proportion was reported for coverage and convergence

^e^(*τ*_0_^2^, *τ*_1_^2^): (Random treatment-effect variance, random study-effect variance)

**Table 7:** Performance of the stratified- and random-intercept^a^ models approaches in large data sets^b^ with greater (Top panel) and lesser (Bottom panel) heterogeneity of random effects^c^

	Performance measures^d^	Data generation			
		Random-study and -treatment effect (Eq. 1)		Stratified-study effect (Eq. 2)	
		Stratified-intercept	Random-intercept	Stratified-intercept	Random-intercept
(*τ*_0_^2^, *τ*_1_^2^) = (4, 4)^e^	AB ($\beta$_1_)	0.04 (0.02, 0.06)	**0.03 (0.02, 0.06)**	0.04 (0.02, 0.06)	0.04 (0.01, 0.06)
	RMSE ($\beta$_1_)	1.11 (0.55, 1.94)	**1.07 (0.49, 1.84)**	1.15 (0.58, 1.98)	**1.11 (0.58, 1.87)**
	Coverage ($\beta$_1_)	**95.2**	92.3	92.3	**93.6**
	AB (*τ*_1_^2^)	0.13 (0.06, 0.20)	**0.12 (0.06, 0.20)**	**0.11 (0.05,0.18)**	0.22 (0.20, 0.25)
	RMSE (*τ*_1_^2^)	4.05 (1.85,6.25)	**3.87 (1.81, 6.21)**	**3.39 (1.57,5.56)**	6.99 (6.20, 7.80)
	Coverage (*τ*_1_^2^)	53.7	**78.9**	**89.8**	1.0
	Convergence	63.8	**98.3**	**99.7**	89.9
(*τ*_0_^2^, *τ*_1_^2^) = (1, 1)	AB ($\beta$_1_)	0.02 (0.01, 0.03)	0.02 (0.01, 0.03)	0.02 (0.01, 0.03)	0.02 (0.01, 0.03)
	RMSE ($\beta$_1_)	0.63 (0.29, 1.07)	**0.59 (0.29, 1.05)**	0.63 (0.30, 1.05)	0.63 (0.30, 1.03)
	Coverage ($\beta$_1_)	91.8	**91.9**	93.1	**93.3**
	AB (*τ*_1_^2^)	0.03 (0.02, 0.06)	0.03 (0.02, 0.05)	0.03 (0.02, 0.05)	**0.02 (0.01, 0.03)**
	RMSE (*τ*_1_^2^)	1.06 (0.52, 1.74)	**1.03 (0.49, 1.68)**	0.98 (0.48, 1.69)	**0.57 (0.27, 1.00)**
	Coverage (*τ*_1_^2^)	**86.3**	82.5	**87.9**	76.5
	Convergence	95.3	**96.5**	**99.2**	88.8

^a^Results are given for Penalized Quasi-likelihood (PQL) for the One-stage random-intercept and random treatment effect model (Model 3) and the stratified-intercept and random-slope model (Model 4)

^b^Large data sets had 15 studies and on average 3000 total subjects

^c^Bold text represent “best value” of performance

^d^Median (25th and 75th percentile) were reported for AB and RMSE, the proportion was reported for coverage and convergence

^e^(*τ*_0_^2^, *τ*_1_^2^): (Random treatment-effect variance, random study-effect variance)

We did not exclude results from meta-analyses that returned a warning message (imperfect convergence). These meta-analyses were included as non-convergence and although these models failed to produce proper parameter estimates, these estimates were included in the calculation of the bias and the MSE.

### One- versus Two-stage

In Tables 2 and 3, results for the absolute bias (AB) of the estimates for the pooled treatment effect *β*_1_ are given. Recalling that the true parameter value was 0.18, we see that the biases were identical and under 0.05 in the one-stage and the two-stage approaches in both small and large data sets. Results were very comparable when the outcome rate was reduced from 30 to 5% (Additional file 1: Table S1). For both the one- and the two-stage, results depended on the true *τ* ^2^, and the sample size.

For the larger sample size, root mean square error (RMSE) in *β*_1_ was generally slightly larger when the one-stage method was used than when the two-stage was used. The picture was similar across all heterogeneity levels (Tables 2 and 3) and when the outcome rate was reduced (Additional file 2: Table S3).

Neither one-stage nor two-stage methods yielded coverage of *β*_1_ close to nominal levels (Tables 2 and 3). Increasing sample size had a positive effect on percent coverage, and increasing the true heterogeneity made estimation more difficult, hence decreasing the coverage (Table 3).

Absolute bias of the between-study heterogeneity, *τ*_1_^2^ was usually slightly lower when the one-stage approach was used than when the two-stage approach was (Tables 2 and 3), particularly, when the sample size was small (Table 2) and when greater amount of heterogeneity exist in the random effects (Bottom panel of Table 2). Regarding the effects of the simulation parameters, AB decreased when data was generated with equal study sizes and increased when the rate of occurrence was reduced (Additional file 3: Table S2). In these cases, the one-stage approach was most biased.

The RMSE of *τ*_1_^2^ for the one-stage estimates was mostly smaller than the RMSE of the two-stage method estimates. For increased sample size or reduction in the level of heterogeneity in the random effects, RMSE of *τ*_1_^2 ^decreased at least by a factor of three across both methods. While the RMSE of *τ*_1_^2 ^was inflated when the outcome rate was reduced, the one-stage method continued to outperform that of the two-stage method (Additional file 4: Table S4).

Convergence was not a problem for the two-stage approach while convergence of the one-stage method varied from 90 to 100% (Tables 2 and 3).

### AGHQ versus PQL

One-stage models estimated via PQL and AGHQ methods often yielded similar AB in *β*_1_. There was no observed difference in the AB (*β*_1_) between the methods when the outcome rate was reduced (Additional file 1: Table S1).

RMSE of *β*_1_ were generally greater when AGHQ was used than when PQL was used (Tables 4 and 5). Decreasing sample size, increasing the variances of the random effects or reducing the event rate (Additional file 2: Table S3) made precise estimation more difficult, hence RMSE increased.

When the true heterogeneity was large and total sample was small (Top panel of Table 4), AGHQ provided coverage for *β*_1_closer to nominal levels than PQL, while both methods provided comparable coverage when the sample size was increased (Table 5). Note that across both methods, levels of coverage were higher as heterogeneity increased and similar coverage was observed when the outcome rate was reduced (Additional file 5: Table S5).

AB in *τ*_1_^2^, was very comparable but slightly lower when PQL was used rather than AGHQ (Tables 4 and 5). The AB decreased with increasing sample size, particularly, when PQL was used (Table 5). There was substantial bias in *τ*_1_^2^ estimates when the event rate was reduced (Additional file 3: Table S2).

On account of a better overall performance of PQL with regards to AB, RMSE of *τ*_1_^2 ^was generally lower with PQL than with AGHQ (Tables 4 and 5). RMSE decreased with decreased variability in the random effects, and with increased sample size. In addition, PQL-estimates continued to yield smaller RMSE than AGHQ-estimates when the outcome rate was reduced (Additional file 4: Table S4).

We found important under-coverage of the estimates for *τ*_1_^2 ^for both estimation methods, particularly when PQL was used (Tables 4 and 5). The percent coverage was usually fair for both estimation methods when sample size increased, but was poor when the outcome rate was reduced (Additional file 6: Table S6).

Convergence occurred more often when AGHQ was used than when PQL was used (Tables 4 and 5). Convergence was problematic for PQL, particularly when true heterogeneity was low and sample size was small (Bottom panel of Table 4). Comparable convergence was seen when the event rate was reduced (Additional file 5: Table S5).

### Random- intercept versus stratified-intercept

The results of the simulation studies, modeling the intercept as random or fixed (random slope was always considered) via PQL estimation are summarized in the Tables 6 and 7.

The convergence was markedly low (14-97%) for the fixed intercept & random slope method (Tables 6 and 7). Convergence was only reasonable for the approach when the sample size was large and heterogeneity was small, whereas convergence was always greater than 80% for the random intercept and slope approach.

In general, AB in *β*_1_ was similar for both stratified-intercept (random-slope only) and random intercept & slope methods. Regarding the simulation parameters, sample size and variability of the random effects, were not influential in reducing the AB in *β*_1_.

The RMSE in *β*_1_ was smaller when estimated via the random intercept and slope model than when only a random slope was fit (Tables 6 and 7).

Increased sample size and level of heterogeneity in the random effect was most influential in determining coverage probability.

Absolute bias in *τ*_1_^2^ was clearly comparable when fit with a random intercept & slope approach or a random slope only (Tables 6 and 7). For lower outcome rate, there was a trend towards less pronounced bias when a random slope only was fit (Additional file 3: Table S2).

We observed lower RMSE of *τ*_1_^2^ when a random intercept was fit, especially when the true heterogeneity was large (Top panels of Tables 6 and 7). Comparable results were seen when both models were fit in large sample and the true heterogeneity was small (Bottom panel of Table 7)- also when outcome rate was reduced (Additional file 4: Table S4).

We found significant under coverage of *τ*_1_^2 ^when both models were fit, however, this was more severe when a random slope only model was fit (Tables 6 and 7). When the generated values of *τ*_0_^2^ or *τ*_1_^2^ were low (i.e. low variability in the random effects) and sample size was increased, we had less difficulty to estimate the coverage of *τ*_1_^2^ when both models were fit. The coverage probability continued to be an issue when the rate of occurrence was reduced (Additional file 6: Table S6).

## Discussion

### Findings

Our simulation results indicate that when the number of subjects per study is large, the one- and two-stage methods yield very similar results. Our results also confirm the finding of previous empirical studies [5, 26, 27] that in some cases, the one-stage and two-stage IPD-MA results coincide. However, we found discrepancies between these methods, with a slight preference towards the one-stage method when the number of subjects per study is small. In these situations, neither method produced accurate estimates for the between-study heterogeneity associated with the treatment-effect; however, the biases were larger for the two-stage approach. Furthermore, one-stage methods produced less biased and more precise estimates of the variance parameter and had slightly higher coverage probabilities, though these differences may be due to using the REML estimate of *τ*_1_^2^ instead of the der Simonian and Laird estimator used in the two-stage approach.

Estimation of GLMMs with binary outcomes continues to pose challenges, with many methods producing biased regression coefficients and variance components [7]. AGHQ has been shown to overestimate the variance component with few clusters or few subjects [17]. On the contrary, PQL has been found to underestimate the variance component while the standard errors are overestimated [12]. In the context of IPD-MA, we found similar absolute bias of the PQL- and AGHQ-estimated pooled treatment effect, while the PQL-estimates of the between-study variance had greater precision when study sizes were small and random effects were correlated. This somewhat confirms previous results, which found that PQL suffers from large biases but performs better in terms of MSE than AGHQ [6]. Both estimation methods experienced difficulty in attaining nominal coverage of the between-study heterogeneity associated with the treatment effect in two situations: (i) when the number of studies included was small and/or (ii) the true variances of the random effects were small. We also found that convergence was not an important problem for AGHQ when meta-analyses included studies with less than 50 individuals per study. However, convergence was poor when the prevalence of the outcome was reduced to 5% and the true heterogeneity was close to zero.

Stratification of the intercept in one-stage models avoids the need to estimate the random effect for the intercept and the correlation between the random effects. This approach may be preferable in situations not investigated in this work (e.g. when the distribution of the random effects is skewed). However, this approach suffered from marked convergence rates when fit to small data sets (15 studies and on average 500 subjects).

### Strengths and Limitations

We used simulation studies to compare various analytic strategies to analyze data arising from IPD-MA across a wide range of data generation scenarios but made some simplifications. We only considered binary outcomes, one dichotomous treatment variable, a two-level data structure, and no confounders. Moreover, we estimated GLMMs via PQL and AGHQ, but did not compare Bayesian or other estimation methods, which might be particularly useful in sparse scenarios [28]. We have made the assumption throughout that IPD were available. Certainly, the time and cost associated with collecting IPD are considerable. However, once such data is in hand, we have addressed several open questions relating to the best way to analyze it. We should also note that methods exist for combining IPD and aggregated data [7]. Further study is needed to investigate alternative confidence intervals (or coverage probability) for the between-study heterogeneity that can be used to remedy the under-coverage of Gaussian intervals. The normality-based intervals (coverage rate) we studied greatly underperformed in most scenarios because the constructions of the confidence interval are likely to be invalid. A further simplification that limits the generalizability of this work is that it is restricted to only two-arm trials. The extension to three or more arms would require careful consideration of more complicated correlation structures in treatment effects across arms and within studies [29].

One important comparison we have not addressed is, computational speed where the two-stage method had a distinct advantage over the one-stage; PQL was faster than AGHQ and the stratified-intercept model run-time was quicker than the random-intercept model.

As far as we know, this simulation study is the first to simultaneously generate data with normally distributed and stratified random intercepts. This study also compares approaches that include a random intercept for study membership to those that do not. Furthermore, the use of simulation - to systematically investigate the robustness of the approaches to variation in sample size, study number, outcome rate, magnitude of correlation and variances. As a result, our scenarios have allowed us to assess performance without being too exhaustive.

### Guidelines for Best Practice

On the basis of these findings, we can make several recommendations. When the IPD-MA included many studies and the outcome rate was not too low, this work supports the conclusion of a previous study [5] that the conventional two-stage method by DerSimonian and Laird [21] is a good choice under the data conditions simulated here. Cornell et al. found that the DL method produced too-narrow confidence bounds and p values that were too small when the number of studies was small or there was high between-study heterogeneity [30]. In such cases, a modification such as the Hartung-Knapp approach may be preferable [31]. Further, while the bivariate two-stage approach is very rarely used in practice, we found that it tended to yield good overall model performance, comparable with that of the one-stage models when study sizes are small. In addition, our results also suggest that the one-stage method can be used in IPD-MA where study sizes are less than 50 subjects per study or few events were recorded in most studies (outcome rate of 5%). In these cases, the one-stage approach is more appropriate as it models the exact binomial distribution of the data and offers more flexibility in model specification over the two-stage approach [32].

If interest lies in estimation of the pooled treatment effect or the between-study heterogeneity of the treatment effect, estimation using PQL appeared to be a better choice due to its lower bias and mean square error for the settings considered. On the contrary, computational issues such as convergence occurred less with this technique than with AGHQ. However, it is important to note that convergence and coverage in *τ* ^2^ was an issue in small and large total sample sizes and also, when level of true heterogeneity was large.

For these simulated data, the results of both the random-intercept and stratified-intercept models were not importantly different. However, under both data generations, fitting a GLMM with the random-intercept was overall less sensitive to misspecification in small sample sizes with large between-study heterogeneity than the stratified-intercept GLMM since we have observed high rates of non-convergence via the stratified-intercept model.

There are four important caveats to these recommendations. First, our simulations show greater accuracy of the pooled odds ratio as the number of studies increase. Therefore, an IPD-MA with more studies will provide more accurate estimates. Secondly, our results show that the estimation of the between-study heterogeneity of the treatment effect is highly biased regardless of the sample size and number of studies. Therefore, we should always expect that the variance parameter be estimated with some error. Thirdly, small overall samples mark the trade-off under which a meta-analyst might consistently choose precision over bias and our simulations show that PQL estimation may be preferred in these situations. Finally, large overall sample size can eliminate the lack of statistical power present in small overall samples. In such cases, comparable results are seen for one- and two-stage methods and fitting a two-stage analysis as a first step may be advisable. This could aid as a quick and efficient investigation of heterogeneity and treatment-outcome association.

## Conclusion

To summarize, the one- and two-stage methods consistently produced similar results when the number of studies and overall sample are large. Although the PQL and AGHQ estimation procedures produced similar bias of the pooled log odds ratios, PQL-estimates had lower RMSE than the AGHQ-estimates. Both the random-intercept and stratified-intercept models yielded precise and similar estimates for the pooled log odds ratios. However, the random-intercept models gave good coverage probabilities of the between-study heterogeneity in small sample sizes and yielded overall good convergence rate as compared to the random slope only model.

## Additional files

Additional file 1: Median (Interquartile range (IQR)) absolute bias (%) for treatment effect, β_1_ for different approach, by number of studies, total average sample size, mixture of studies sizes and degree of random effects variances - data generated from random study- and treatment effect: Eq. 1 with 5% outcome rate. (DOC 72 kb)
Additional file 2: Median (Interquartile range (IQR)) (%) root mean square error for treatment effect, β_1_ for different approach, by number of studies, total average sample size, mixture of studies sizes and degree of random effects variances - data generated from random study- and treatment effect: Eq. 1 with 5% outcome rate. (DOC 70 kb)
Additional file 3: Median (Interquartile range (IQR)) absolute bias (%) for random treatment-effect variance, τ^2^_1_ for different approach, by number of studies, total average sample size, mixture of studies sizes and degree of random effects variances - data generated from random study- and treatment effect: Eq. 1 with 5% outcome rate. (DOC 73 kb)
Additional file 4: Median (Interquartile range (IQR)) (%) root mean square error for random treatment-effect variance, τ^2^_1_ for different approach, by number of studies, total average sample size, mixture of studies sizes and degree of random effects variances - data generated from random study- and treatment effect: Eq. 1 with 5% outcome rate. (DOC 63 kb)
Additional file 5: Percent Coverage (percent convergence rate) for treatment effect, β_1_ for different approach, by number of studies, total average sample size, mixture of studies sizes and degree of random effects variances - data generated from random study- and treatment effect: Eq. 1 with 5% outcome rate. (DOC 62 kb)
Additional file 6: Percent Coverage for random treatment-effect variance, τ^2^_1_ for different approach, by number of studies, total average sample size, mixture of studies sizes and degree of random effects variances - data generated from random study- and treatment effect: Eq. 1 with 5% outcome rate. (DOC 63 kb)

## Abbreviations

AB: Absolute bias
AGHQ: Adaptive Gaussian hermite quadrature
GLMM: Generalized linear mixed model
IPD-MA: Individual patient data meta-analysis
MOM: Method-of-moments
MSE: Mean-squared error
PQL: Penalized quasi-likelihood
REML: Restricted maximum likelihood
