# Supplementary material for: A comparison of analytic approaches for individual patient data meta-analyses with binary outcomes
Source: BMC Med Res Methodol. 2017 Feb 16;17:28. doi: 10.1186/s12874-017-0307-7 (PMC5312561; doi:10.1186/s12874-017-0307-7)
Supplement: Additional file 1: — Median (Interquartile range (IQR)) absolute bias (%) for treatment effect, β1 for different approach, by number of studies, total average sample size, mixture of studies sizes and degree of random effects variances - data generated from random study- and treatment effect: Eq. 1 with 5% outcome rate. (DOC 72 kb) [file 12874_2017_307_MOESM1_ESM.doc]

Table S1: Median (25^th^ and 75^th^ centiles) absolute bias (%)[[1]](#footnote-2) for treatment effect, β_1_ for different approach, by number of studies, total average sample size, mixture of studies sizes and degree of random effects variances (data generated from random study- and treatment effect: Equation 1 with 5% outcome rate)

|  |  | Equally sized | | | | | | 25% large studies | | | | | | | | |
| --- | --- | --- | --- | --- | --- | --- | --- | --- | --- | --- | --- | --- | --- | --- | --- | --- |
|  |  | Random-effects Variances (τ^2^_0_, τ^2^_1_)[[2]](#footnote-3) | | | | | | Random-effects Variances (τ^2^_0_, τ^2^_1_) | | | | | | | | |
| (Number of studies, total average sample size) | Methods[[3]](#footnote-4) | (0.05, 0.05) | (0.05, 1) | (0.05, 4) | (1,1) | (1,4) | (4,4) | (0.05, 0.05) | (0.05, 1) | | (0.05, 4) | (1,1) | (1,4) | | (4,4) | |
| (5,500) | Model 1 | 0.027 (0.011, 0.048) | 0.039 (0.020, 0.066) | 0.058 (0.026, 0.101) | 0.037 (0.018, 0.064) | 0.056 (0.028, 0.092) | 0.054 (0.024, 0.096) | 0.029 (0.013, 0.051) | 0.044 (0.021, 0.074) | | 0.062 (0.028, 0.105) | 0.042 (0.020, 0.073) | 0.059 (0.030, 0.099) | | 0.057 (0.027, 0.097) | |
|  | Model 2 | 0.026 (0.012, 0.045) | 0.037 (0.018, 0.060) | 0.055 (0.025, 0.097) | 0.039 (0.019, 0.064) | 0.060 (0.030, 0.098) | 0.065 (0.030, 0.108) | 0.029 (0.014, 0.049) | 0.046 (0.022, 0.074) | | 0.060 (0.029, 0.103) | 0.046 (0.023, 0.074) | 0.060 (0.030, 0.103) | | 0.062 (0.031, 0.104) | |
|  | Model 3 (PQL) | 0.030 (0.013, 0.053) | 0.043 (0.020, 0.074) | 0.066 (0.031, 0.116) | 0.043 (0.020, 0.077) | 0.067 (0.034, 0.109) | 0.070 (0.031, 0.125) | 0.031 (0.014, 0.058) | 0.052 (0.034, 0.132) | | 0.075 (0.033, 0.132) | 0.050 (0.023, 0.090) | 0.077 (0.038, 0.129) | | 0.079 (0.035, 0.135) | |
|  | Model 3(AGHQ) | 0.029 (0.012, 0.049) | 0.041 (0.018, 0.067) | 0.066 (0.025, 0.118) | 0.043 (0.021, 0.079) | 0.074 (0.036, 0.123) | 0.065 (0.031, 0.136) | 0.027 (0.013, 0.049) | 0.046 (0.021, 0.082) | | 0.073 (0.034, 0.130) | 0.051 (0.024, 0.091) | 0.076 (0.039, 0.147) | | 0.094 (0.043, 0.155) | |
|  | Model 4 (PQL) | 0.029 (0.013, 0.054) | 0.046 (0.022, 0.077) | 0.070 (0.031, 0.124) | 0.048 (0.022, 0.078) | 0.071 (0.037, 0.113) | 0.071 (0.033, 0.125) | 0.033 (0.014, 0.056) | 0.051 (0.022, 0.086) | | 0.068 (0.032, 0.123) | 0.045 (0.020, 0.092) | 0.067 (0.036, 0.119) | | 0.084 (0.041, 0.131) | |
|  | Model 4 (AGHQ) | 0.029 (0.013, 0.054) | 0.045 (0.022, 0.075) | 0.068 (0.030, 0.019) | 0.046 (0.022, 0.080) | 0.072 (0.037, 0.116) | 0.077 (0.037, 0.134) | 0.031 (0.015, 0.056) | 0.049 (0.023, 0.088) | | 0.074 (0.034, 0.134) | 0.052 (0.025, 0.091) | 0.080 (0.039, 0.146) | | 0.085 (0.039, 0.141) | |
| (15, 3000) | Model 1 | 0.013 (0.006, 0.021) | 0.019 (0.010, 0.032) | 0.032 (0.015, 0.055) | 0.019 (0.010, 0.032) | 0.030 (0.015, 0.052) | 0.031 (0.014, 0.053) | 0.013 (0.007, 0.021) | 0.022 (0.011, 0.039) | 0.035 (0.016, 0.062) | | 0.022 (0.011, 0.039) | | 0.035 (0.016, 0.059) | | 0.035 (0.017, 0.058) |
|  | Model 2 | 0.011 (0.006, 0.020) | 0.019 (0.009, 0.033) | 0.033 (0.016, 0.057) | 0.024 (0.011, 0.040) | 0.034 (0.016, 0.059) | 0.039 (0.018, 0.066) | 0.012 (0.006, 0.021) | 0.022 (0.011, 0.039) | 0.033 (0.015, 0.058) | | 0.025 (0.012, 0.042) | | 0.036 (0.017, 0.063) | | 0.042 (0.021, 0.068) |
|  | Model 3 (PQL) | 0.013 (0.007, 0.022) | 0.020 (0.009, 0.034) | 0.038 (0.018, 0.062) | 0.022 (0.010, 0.036) | 0.034 (0.016, 0.057) | 0.036 (0.016, 0.060) | 0.014 (0.007, 0.022) | 0.024 (0.011, 0.044) | 0.041 (0.020, 0.069) | | 0.025 (0.012, 0.044) | | 0.039 (0.017, 0.067) | | 0.041 (0.020, 0.069) |
|  | Model 3(AGHQ) | 0.015 (0.008, 0.022) | 0.022 (0.010, 0.036) | 0.037 (0.019, 0.065) | 0.023 (0.011, 0.038) | 0.038 (0.018, 0.065) | 0.041 (0.020, 0.072) | 0.013 (0.007, 0.022) | 0.026 (0.012, 0.048) | 0.041 (0.019, 0.075) | | 0.026 (0.013, 0.045) | | 0.042 (0.022, 0.074) | | 0.046 (0.023, 0.083) |
|  | Model 4 (PQL) | 0.013 (0.007, 0.022) | 0.021 (0.010, 0.036) | 0.038 (0.018, 0.064) | 0.025 (0.012, 0.042) | 0.035 (0.017, 0.062) | 0.038 (0.017, 0.065) | 0.013 (0.006, 0.022) | 0.024 (0.012, 0.043) | 0.039 (0.018, 0.070) | | 0.026 (0.013, 0.044) | | 0.043 (0.016, 0.071) | | 0.049 (0.018, 0.074) |
|  | Model 4 (AGHQ) | 0.013 (0.007, 0.022) | 0.021 (0.010, 0.036) | 0.037 (0.018, 0.064) | 0.025 (0.011, 0.041) | 0.036 (0.018, 0.063) | 0.042 (0.020, 0.074) | 0.013 (0.007, 0.022) | 0.025 (0.011, 0.044) | 0.041 (0.018, 0.070) | | 0.028 (0.014, 0.047) | | 0.043 (0.019, 0.075) | | 0.052 (0.026, 0.085) |
| (50,9000) | Model 1 | 0.007 (.003, 0.011) | 0.010 (0.005, 0.018) | 0.019 (0.008, 0.030) | 0.010 (0.005, 0.019) | 0.018 (0.008, 0.030) | 0.019 (0.009, 0.032) | 0.008 (0.004, 0.013) | 0.013 (0.006, 0.022) | | 0.020 (0.009, 0.032) | 0.014 (0.007, 0.023) | 0.020 (0.010, 0.035) | | 0.021 (0.010, 0.036) | |
|  | Model 2 | 0.006 (0.003, 0.011) | 0.011 (0.005, 0.018) | 0.018 (0.009, 0.031) | 0.018 (0.009, 0.031) | 0.023 (0.010, 0.039) | 0.034 (0.018, 0.051) | 0.008 (0.003, 0.013) | 0.013 (0.006, 0.021) | | 0.019 (0.009, 0.031) | 0.017 (0.008, 0.028) | 0.024 (0.010, 0.041) | | 0.032 (0.017, 0.050) | |
|  | Model 3 (PQL) | 0.007 (0.003, 0.011) | 0.012 (0.005, 0.020) | 0.022 (0.009, 0.033) | 0.012 (0.005, 0.021) | 0.019 (0.009, 0.034) | 0.020 (0.010, 0.034) | 0.009 (0.004, 0.014) | 0.013 (0.007, 0.024) | | 0.021 (0.010, 0.038) | 0.013 (0.006, 0.024) | 0.023 (0.012, 0.040) | | 0.022 (0.011, 0.038) | |
|  | Model 3(AGHQ) | 0.008 (0.004, 0.013) | 0.012 (0.005, 0.020) | 0.021 (0.009, 0.035) | 0.013 (0.006, 0.021) | 0.022 (0.010, 0.037) | 0.022 (0.010, 0.037) | 0.008 (0.004, 0.014) | 0.014 (0.007, 0.025) | | 0.024 (0.012, 0.041) | 0.015 (0.007, 0.025) | 0.024 (0.011, 0.042) | | 0.026 (0.012, 0.044) | |
|  | Model 4 (PQL) | 0.008 (0.004, 0.013) | 0.012 (0.006, 0.020) | 0.020 (0.009, 0.035) | 0.015 (0.007, 0.025) | 0.022 (0.010, 0.039) | 0.024 (0.007, 0.039) | 0.009 (0.003, 0.013) | 0.014 (0.007, 0.025) | | 0.021 (0.010, 0.034) | 0.035 (0.009, 0.036) | 0.052 (0.040, 0.065) | | 0.049 (0.018, 0.074) | |
|  | Model 4 (AGHQ) | 0.007 (0.004, 0.012) | 0.012 (0.006, 0.020) | 0.021 (0.009, 0.035) | 0.017 (0.008, 0.028) | 0.023 (0.011, 0.040) | 0.034 (0.017, 0.053) | 0.009 (0.004, 0.014) | 0.014 (0.007, 0.024) | | 0.022 (0.010, 0.038) | 0.019 (0.009, 0.033) | 0.029 (0.013, 0.049) | | 0.052 (0.026, 0.085) | |

1. Absolute relative percent bias of β_1_ was calculated for each simulated meta-analysis first, and then summarized across meta-analyses. For each combination of data generation parameters, 1000 meta-analyses were generated. [↑](#footnote-ref-2)
2. τ^2^_0_ is the random study-effect variance and τ^2^_1_, the random treatment-effect variance [↑](#footnote-ref-3)
3. Model 1 (bivariate two-stage); Model 2 (conventional DerSimonian and Laird two-stage); Model 3 (random intercept and random slope one-stage via PQL and AGHQ); Model 4 (stratified intercept one-stage via PQL and AGHQ). [↑](#footnote-ref-4)
