# Supplementary material for: A comparison of analytic approaches for individual patient data meta-analyses with binary outcomes
Source: BMC Med Res Methodol. 2017 Feb 16;17:28. doi: 10.1186/s12874-017-0307-7 (PMC5312561; doi:10.1186/s12874-017-0307-7)
Supplement: Additional file 2: — Median (Interquartile range (IQR)) (%) root mean square error for treatment effect, β1 for different approach, by number of studies, total average sample size, mixture of studies sizes and degree of random effects variances - data generated from random study- and treatment effect: Eq. 1 with 5% outcome rate. (DOC 70 kb) [file 12874_2017_307_MOESM2_ESM.doc]

**Table S3: Median (Interquartile range (IQR)) (%) root mean square error[[1]](#footnote-2) for treatment effect, β_1_ for different approach, by number of studies, total average sample size, mixture of studies sizes and degree of random effects variances (data generated from random study- and treatment effect: Equation 1 with 5% outcome rate)**

|  |  | Equally sized | | | | | | 25% large studies | | | | | | | | |
| --- | --- | --- | --- | --- | --- | --- | --- | --- | --- | --- | --- | --- | --- | --- | --- | --- |
|  |  | Random-effects Variances (τ^2^_0_, τ^2^_1_)[[2]](#footnote-3) | | | | | | Random-effects Variances (τ^2^_0_, τ^2^_1_) | | | | | | | | |
| (Number of studies, total average sample size) | Methods[[3]](#footnote-4) | (0.05, 0.05) | (0.05, 1) | (0.05, 4) | (1,1) | (1,4) | (4,4) | (0.05, 0.05) | (0.05, 1) | | (0.05, 4) | (1,1) | (1,4) | | (4,4) | |
| (5,500) | Model 1 | 0.844 (0.353, 1.531) | 1.246 (0.636, 2.082) | 1.837 (0.817, 3.195) | 1.172 (0.557, 2.010) | 1.785 (0.887, 2.911) | 1.708 (0.759, 3.029) | 0.917 (0.424, 1.603) | 1.398 (0.658, 2.346) | | 1.945 (0.897, 3.316) | 1.322 (0.631, 2.310) | 1.872 (0.960, 3.126) | | 1.812 (0.852, 3.064) | |
|  | Model 2 | 0.830 (0.365, 1.437) | 1.176 (0.582, 1.908) | 1.733 (0.787, 3.077) | 1.248 (0.588, 2.027) | 1.885 (0.963, 3.086) | 2.044 (0.958, 3.425) | 0.923 (0.430, 1.537) | 1.442 (0.708, 2.343) | | 1.897 (0.914, 3.259) | 1.447 (0.726, 2.353) | 1.894 (0.961, 3.259) | | 1.967 (0.993, 3.300) | |
|  | Model 3 (PQL) | 0.937 (0.396, 1.671) | 1.363 (0.642, 2.328) | 2.103 (0.986, 3.672) | 1.372 (0.632, 2.424) | 2.106 (1.062, 3.434) | 2.207 (0.970, 3.956) | 0.988 (0.444, 1.824) | 1.650 (0.732, 2.843) | | 2.379 (1.073, 4.171) | 1.569 (0.743, 2.853) | 2.438 (1.216, 4.067) | | 2.489 (1.116, 4.278) | |
|  | Model 3(AGHQ) | 0.902 (0.381, 1.565) | 1.281 (0.581, 2.132) | 2.097 (0.804, 3.737) | 1.375 (0.666, 2.510) | 2.327 (1.146, 3.875) | 2.051 (0.995, 4.291) | 0.867 (0.417, 1.547) | 1.463 (0.663, 2.600) | | 2.300 (1.087, 4.100) | 1.598 (0.762, 2.867) | 2.410 (1.235, 4.658) | | 2.976 (1.355, 4.888) | |
|  | Model 4 (PQL) | 0.930 (0.413, 1.718) | 1.468 (0.709, 2.440) | 2.206 (0.977, 3.928) | 1.505 (0.706, 2.470) | 2.252 (1.160, 3.575) | 2.243 (1.058, 3.953) | 1.053 (0.459. 1/746) | 1.607 (0.702, 2.731) | | 2.137 (0.999, 3.895) | 1.417 (0.636, 2.595) | 2.127 (1.126, 3.756) | | 2.661 (1.293, 4.150) | |
|  | Model 4 (AGHQ) | 0.930 (0.402, 1.700) | 1.432 (0.691, 2.372) | 2.141 (0.939, 3.754) | 1.440 (0.698, 2.516) | 2.284 (1.165, 3.676) | 2.428 (1.157, 4.237) | 1.000 (0.462, 1.777) | 1.555 (0.736, 2.788) | | 2.348 (1.083, 4.240) | 1.651 (0.780, 2.889) | 2.527 (1.237, 4.608) | | 2.672 (1.248, 4.463) | |
| (15, 3000) | Model 1 | 0.402 (0.190, 0.648) | 0.598 (0.300, 1.006) | 1.010 (0.487, 1.744) | 0.596 (0.311,0.998) | 0.960 (0.462, 1.641) | 0.980 (0.454, 1.679) | 0.400 (0.209, 0.655) | 0.696 (0.335, 1.219) | 1.104 (0.500, 1.956) | | 0.683 (0.338, 1.241) | | 1.107 (0.506, 1.864) | | 1.099 (0.544, 1.829) |
|  | Model 2 | 0.370 (0.201, 0.640) | 0.596 (0.299, 1.036) | 1.031 (0.498, 1.807) | 0.751 (0.346, 1.271) | 1.074 (0.512, 1.863) | 1.238 (0.572, 2.098) | 0.376 (0.187, 0.655) | 0.700 (0.335, 1.225) | 1.032 (0.466, 1.848) | | 0.781 (0.384, 1.312) | | 1.140 (0.536, 1.997) | | 1.334 (0.650, 2160) |
|  | Model 3 (PQL) | 0.414 (0.223, 0.687) | 0.625 (0.288, 1.065) | 1.203 (0.554, 1.949) | 0.687 (0.327, 1.132) | 1.070 (0.501, 0.564) | 1.150 (0.519, 1.907) | 0.437 (0.208, 0.710) | 0.757 (0.356, 1.387) | 1.292 (0.622, 2.180) | | 0.786 (0.376, 1.394) | | 1.234 (0.542, 2.124) | | 1.311 (0.630, 2.197) |
|  | Model 3(AGHQ) | 0.461 (0.245, 0.701) | 0.684 (0.324, 1.142) | 1.172 (0.609, 2.044) | 0.721 (0.344, 1.211) | 1.206 (0.564, 2.055) | 1.288 (0.640, 2.272) | 0.423 (0.206, 0.709) | 0.820 (0.384, 1.505) | 1.306 (0.590, 2.367) | | 0.808 (0.396, 1.415) | | 1.318 (0.703, 2.354) | | 1.452 (0.713, 2.614) |
|  | Model 4 (PQL) | 0.410 (0.212, 0.693) | 0.678 (0.321, 1.133) | 1.186 (0.565, 2.036) | 0.790 (0.381, 1.323) | 1.116 (0.529, 1.975) | 1.210 (0.545, 2.069) | 0.414 (0.199, 0.705) | 0.755 (0.365, 1.344) | 1.221 (0.580, 2.199) | | 0.814 (0.399, 1.396) | | 1.360 (0.505, 2.251) | | 1.541 (0.567, 2.334) |
|  | Model 4 (AGHQ) | 0.412 (0.211, 0.689) | 0.670 (0.317, 1.132) | 1.176 (0.558, 2.015) | 0.783 (0.349, 1.307) | 1.145 (0.556, 2) | 1.320 (0.643, 2.345) | 0.425 (0.220, 0.707) | 0.781 (0.349, 1.377) | 1.297 (0.579, 2.201) | | 0.892 (0.428, 1.473) | | 1.349 (0.596, 2.382) | | 1.638 (0.819, 2.696) |
| (50,9000) | Model 1 | 0.206 (0.101, 0.357) | 0.319 (0.145, 0.557) | 0.571 (0.255, 0.938) | 0.321 (0.152, 0.587) | 0.566 (0.261, 0.947) | 0.610 (0.291, 1.015) | 0.247 (0.114, 0.411) | 0.402 (0.198, 0.693) | | 0.629 (0.278, 1.015) | 0.433 (0.212, 0.732) | 0.634 (0.300, 1.118) | | 0.655 (0.310, 1.127) | |
|  | Model 2 | 0.202 (0.098, 0.338) | 0.348 (0.155, 0.568) | 0.572 (0.272, 0.974) | 0.579 (0.285, 0.914) | 0.734 (0.323, 1.222) | 1.075 (0.561, 1.613) | 0.240 (0.108, 0.405) | 0.402 (0.199, 0.677) | | 0.615 (0.278, 0.969) | 0.541 (0.267, 0.873) | 0.773 (0.316, 1.311) | | 1.020 (0.538, 1.576) | |
|  | Model 3 (PQL) | 0.206 (0.082, 0.344) | 0.383 (0.162, 0.618) | 0.687 (0.276, 1.053) | 0.369 (0.164, 0.657) | 0.609 (0.282, 1.060) | 0.623 (0.310, 1.070) | 0.275 (0.127, 0.442) | 0.425 (0.207, 0.764) | | 0.668 (0.315, 1.204) | 0.414 (0.190, 0.754) | 0.736 (0.365, 1.273) | | 0.695 (0.338, 1.193) | |
|  | Model 3(AGHQ) | 0.244 (0.112, 0.398) | 0.385 (0.169, 0.620) | 0.676 (0.299, 1.102) | 0.401 (0.193, 0.676) | 0.687 (0.324, 1.177) | 0.683 (0.303, 1.155) | 0.261 (0.128, 0.450) | 0.455 (0.231, 0.796) | | 0.759 (0.370, 1.309) | 0.481 (0.216, 0.789) | 0.753 (0.355, 1.329) | | 0.826 (0.378, 1.390) | |
|  | Model 4 (PQL) | 0.241 (0.113, 0.409) | 0.388 (0.181, 0.631) | 0.648 (0.298, 1.098) | 0.476 (0.213, 0.777) | 0.689 (0.314, 1.227) | 0.763 (0.235, 1.218) | 0.271 (0.110, 0.397) | 0.443 (0.231, 0.782) | | 0.665 (0.307, 1.073) | 1.109 (0.294, 1.126) | 1.643 (1.255, 2.068) | | 1.541 (0.567, 2.334) | |
|  | Model 4 (AGHQ) | 0.235 (0.115, 0.387) | 0.388 (0.182, 0.633) | 0.649 (0.297, 1.096) | 0.537 (0.252, 0.898) | 0.734 (0.339, 1.250) | 1.062 (0.542, 1.676) | 0.277 (0.131, 0.436) | 0.458 (0.215, 0.756) | | 0.704 (0.329, 1.205) | 0.608 (0.293, 1.032) | 0.904 (0.398, 1.545) | | 1.638 (0.819, 2.696) | |

1. Percent root mean square error of β_1_ was calculated for each simulated meta-analysis first, and then summarized across meta-analyses. For each combination of data generation parameters, 1000 meta-analyses were generated. [↑](#footnote-ref-2)
2. τ^2^_0_ is the random study-effect variance and τ^2^_1_, the random treatment-effect variance [↑](#footnote-ref-3)
3. Model 1 (bivariate two-stage); Model 2 (conventional DerSimonian and Laird two-stage); Model 3 (random intercept and random slope one-stage via PQL and AGHQ); Model 4 (stratified intercept one-stage via PQL and AGHQ). [↑](#footnote-ref-4)
