# Supplementary material for: A comparison of analytic approaches for individual patient data meta-analyses with binary outcomes
Source: BMC Med Res Methodol. 2017 Feb 16;17:28. doi: 10.1186/s12874-017-0307-7 (PMC5312561; doi:10.1186/s12874-017-0307-7)
Supplement: Additional file 3: — Median (Interquartile range (IQR)) absolute bias (%) for random treatment-effect variance, τ2 1 for different approach, by number of studies, total average sample size, mixture of studies sizes and degree of random effects variances - data generated from random study- and treatment effect: Eq. 1 with 5% outcome rate. (DOC 73 kb) [file 12874_2017_307_MOESM3_ESM.doc]

Table S1: Median (Interquartile range (IQR)[[1]](#footnote-2)) absolute bias (%)[[2]](#footnote-3) for random treatment-effect variance, τ^2^_1_ for different approach, by number of studies, total average sample size, mixture of studies sizes and degree of random effects variances (data generated from random study- and treatment effect: Equation 1 with 5% outcome rate)

|  |  | Equally sized | | | | | | 25% large studies | | | | | | | | |
| --- | --- | --- | --- | --- | --- | --- | --- | --- | --- | --- | --- | --- | --- | --- | --- | --- |
|  |  | Random-effects Variances (τ^2^_0_, τ^2^_1_)[[3]](#footnote-4) | | | | | | Random-effects Variances (τ^2^_0_, τ^2^_1_) | | | | | | | | |
| (Number of studies, total average sample size) | Methods[[4]](#footnote-5) | (0.05, 0.05) | (0.05, 1) | (0.05, 4) | (1,1) | (1,4) | (4,4) | (0.05, 0.05) | (0.05, 1) | | (0.05, 4) | (1,1) | (1,4) | | (4,4) | |
| (5,500) | Model 1 | 0.005 (0.005, 0.012) | 0.080 (0.043, 0.100) | 0.234 (0.122, 0.339) | 0.075 (0.041, 0.099) | 0.260 (0.137, 0.354) | 0.248 (0.130, 0.353) | 0.005 (0.005, 0.015) | 0.091 (0.053, 0.100) | | 0.273 (0.146, 0.372) | 0.084 (0.047, 0.100) | 0.283 (0.156, 0.380) | | 0.296 (0.178, 0.376) | |
|  | Model 2 | 0.005 (0.005, 0.005) | 0.100 (0.064, 0.100) | 0.241 (0.119, 0.400) | 0.100 (0.058, 0.100) | 0.270 (0.140, 0.400) | 0.269 (0.142, 0..400) | 0.005 (0.005, 0.005) | 0.100 (0.100, 0.100) | | 0.326 (0.183, 0.400) | 0.100 (0.100, 0.100) | 0.314 (0.173, 0.400) | | 0.327 (0.189, 0.400) | |
|  | Model 3 (PQL) | 0.005 (0.005, 0.005) | 0.100 (0.100, 0.100) | 0.400 (0.400, 0.400) | 0.100 (0.100, 0.100) | 0.400 (0.400, 0.400) | 0.400 (0.271, 0.400) | 0.005 (0.005, 0.005) | 0.100 (0.100, 0.100) | | 0.400 (0.400, 0.400) | 0.100 (0.100, 0.100) | 0.400 (0.400, 0.400) | | 0.400 (0.400, 0.400) | |
|  | Model 3(AGHQ) | 0.033 (0.005, 0.076) | 0.062 (0.029, 0.098) | 0.188 (0.092, 0.289) | 0.074 (0.041, 0.096) | 0.252 (0.127, 0.348) | 0.283 (0.157, 0.377) | 0.005 (0.005, 0.018) | 0.081 (0.054, 0.097) | | 0.329 (0.243, 0.376) | 0.086 (0.061, 0.098) | 0.337 (0.244, 0.386) | | 0.360 (0.259, 0.396) | |
|  | Model 4 (PQL) | 0.005 (0.005, 0.034) | 0.093 (0.042, 0.100) | 0.248 (0.134, 0.388) | 0.096 (0.050, 0.100) | 0.252 (0.127, 0.386) | 0.246 (0.130, 0.378) | 0.005 (0.005, 0.069) | 0.100 (0.069, 0.128) | | 0.294 (0.138, 0.400) | 0.100 (0.066, 0.116) | 0.299 (0.159, 0.400) | | 0.311 (0.161, 0.400) | |
|  | Model 4 (AGHQ) | 0.005 (0.005, 0.005) | 0.098 (0.050, 0.100) | 0.245 (0.132, 0.351) | 0.100 (0.055, 0.100) | 0.266 (0.135, 0.376) | 0.279 (0.146, 0.397) | 0.005 (0.005, 0.005) | 0.100 (0.094, 0.100) | | 0.327 (0.172, 0.400) | 0.100 (0.089, 0.100) | 0.332 (0.163, 0.400) | | 0.352 (0.197, 0.400) | |
| (15, 3000) | Model 1 | 0.005 (0.003, 0.005) | 0.041 (0.020, 0.065) | 0.136 (0.067, 0.208) | 0.043 (0.022, 0.067) | 0.155 (0.080, 0.225) | 0.152 (0.084, 0.230) | 0.005 (0.003, 0.006) | 0.045 (0.023, 0.069) | 0.133 (0.068, 0.208) | | 0.050 (0.026, 0.073) | | 0.184 (0.100, 0.257) | | 0.191 (0.107, 0.270) |
|  | Model 2 | 0.005 (0.005, 0.005) | 0.048 (0.025, 0.071) | 0.140 (0.066, 0.211) | 0.045 (0.022, 0.068) | 0.149 (0.076, 0.220) | 0.150 (0.086, 0.227) | 0.005 (0.005, 0.005) | 0.053 (0.028, 0.077) | 0.195 (0.115, 0.261) | | 0.052 (0.026, 0.075) | | 0.192 (0.107, 0.264) | | 0.194 (0.107, 0.268) |
|  | Model 3 (PQL) | 0.005 (0.005, 0.005) | 0.100 (0.100, 0.100) | 0.400 (0.400, 0.400) | 0.100 (0.100, 0.100) | 0.263 (0.230, 0.400) | 0.253 (0.227, 0.282) | 0.005 (0.005, 0.005) | 0.100 (0.100, 0.100) | 0.400 (0.400, 0.400) | | 0.100 (0.052, 0.100) | | 0.295 (0.241, 0.400) | | 0.265 (0.230, 0.309) |
|  | Model 3(AGHQ) | 0.007 (0.004, 0.024) | 0.033 (0.015, 0.058) | 0.107 (0.051, 0.177) | 0.040 (0.019, 0.067) | 0.117 (0.057, 0.199) | 0.143 (0.072, 0.221) | 0.005 (0.003, 0.013) | 0.047 (0.023, 0.071) | 0.143 (0.069, 0.229) | | 0.047 (0.022, 0.076) | | 0.147 (0.074, 0.239) | | 0.144 (0.069, 0.249) |
|  | Model 4 (PQL) | 0.005 (0.005, 0.012) | 0.036 (0.019, 0.062) | 0.126 (0.065, 0.206) | 0.039 (0.021, 0.065) | 0.117 (0.053, 0.192) | 0.130 (0.065, 0.201) | 0.005 (0.005, 0.007) | 0.046 (0.021, 0.071) | 0.125 (0.064, 0.210) | | 0.045 (0.022, 0.071) | | 0.142 (0.069, 0.224) | | 0.138 (0.067, 0.228) |
|  | Model 4 (AGHQ) | 0.005 (0.005, 0.007) | 0.037 (0.018, 0.060) | 0.129 (0.059, 0.202) | 0.040 (0.019, 0.062) | 0.127 (0.059, 0.196) | 0.136 (0.066, 0.206) | 0.005 (0.005, 0.005) | 0.046 (0.023, 0.072) | 0.141 (0.073, 0.218) | | 0.046 (0.023, 0.070) | | 0.146 (0.079, 0.226) | | 0.149 (0.075, 0.229) |
| (50,9000) | Model 1 | 0.004 (0.002, 0.005) | 0.032 (0.018, 0.047) | 0.088 (0.048, 0.136) | 0.036 (0.019, 0.052) | 0.143 (0.092, 0.184) | 0.152 (0.107, 0.195) | 0.004 (0.002, 0.005) | 0.029 (0.014, 0.046) | | 0.104 (0.056, 0.152) | 0.042 (0.024, 0.056) | 0.169 (0.125, 0.212) | | 0.190 (0.151, 0.228) | |
|  | Model 2 | 0.005 (0.005, 0.005) | 0.047 (0.031, 0.062) | 0.132 (0.082, 0.176) | 0.036 (0.019, 0.052) | 0.138 (0.089, 0.178) | 0.150 (0.102, 0.192) | 0.005 (0.003, 0.005) | 0.045 (0.028, 0.060) | | 0.194 (0.149, 0.229) | 0.043 (0.028, 0.059) | 0.188 (0.145, 0.227) | | 0.187 (0.147, 0.227) | |
|  | Model 3 (PQL) | 0.005 (0.004, 0.006) | 0.031 (0.014, 0.076) | 0.065 (0.031, 0.112) | 0.023 (0.011, 0..038) | 0.082 (0.043, 0.127) | 0.089 (0.048, 0.137) | 0.005 (0.005, 0.005) | 0.100 (0.100, 0.100) | | 0.400 (0.232, 0.400) | 0.047 (0.017, 0.100) | 0.249 (0.233, 0.269) | | 0.248 (0.235, 0.263) | |
|  | Model 3(AGHQ) | 0.005 (0.003, 0.011) | 0.021 (0.010, 0.038) | 0.060 (0.025, 0.104) | 0.023 (0.011, 0.038) | 0.066 (0.034, 0.117) | 0.073 (0.035, 0.124) | 0.004 (0.002, 0.008) | 0.027 (0.013, 0.044) | | 0.077 (0.038, 0.128) | 0.026 (0.012, 0.044) | 0.078 (0.036, 0.132) | | 0.079 (0.036, 0.134) | |
|  | Model 4 (PQL) | 0.005 (0.003, 0.007) | 0.021 (0.010, 0.035) | 0.068 (0.032, 0112) | 0.022 (0.011, 0.038) | 0.066 (0.031, 0.113) | 0.079 (0.038, 0.126) | 0.005 (0.003, 0.005) | 0.026 (0.012, 0.043) | | 0.076 (0.038, 0.122) | 0.025 (0.011, 0.041 | 0.076 (0.038, 0.122) | |  | |
|  | Model 4 (AGHQ) | 0.005 (0.003, 0.005) | 0.022 (0.011, 0.036) | 0.074 (0.037, 0.119) | 0.024 (0.011, 0.038) | 0.082 (0.040, 0.126) | 0.089 (0.048, 0.138) | 0.005 (0.003, 0.005) | 0.026 (0.012, 0.042) | | 0.097 (0.045, 0.143) | 0.025 (0.012, 0.042 | 0.097 (0.045, 0.143) | |  | |

1. IQR reported as (first quartile, Q_1_ and third quartile, Q_3_). [↑](#footnote-ref-2)
2. Median absolute percent bias of beta1 was calculated for each scenario first, and then summarized across scenarios. For each combination of data generation parameters, 1000 meta-analyses were generated. [↑](#footnote-ref-3)
3. τ^2^_0_ is the random study-effect variance and τ^2^_1_, the random treatment-effect variance [↑](#footnote-ref-4)
4. Model 1 (bivariate two-stage); Model 2 (conventional DerSimonian and Laird two-stage); Model 3 (random intercept and random slope one-stage via PQL and AGHQ); Model 4 (stratified intercept one-stage via PQL and AGHQ). [↑](#footnote-ref-5)
