# Supplementary material for: A comparison of analytic approaches for individual patient data meta-analyses with binary outcomes
Source: BMC Med Res Methodol. 2017 Feb 16;17:28. doi: 10.1186/s12874-017-0307-7 (PMC5312561; doi:10.1186/s12874-017-0307-7)
Supplement: Additional file 4: — Median (Interquartile range (IQR)) (%) root mean square error for random treatment-effect variance, τ2 1 for different approach, by number of studies, total average sample size, mixture of studies sizes and degree of random effects variances - data generated from random study- and treatment effect: Eq. 1 with 5% outcome rate. (DOC 63 kb) [file 12874_2017_307_MOESM4_ESM.doc]

**Table S4**

**: Median (Interquartile range (IQR)) (%) root mean square error[[1]](#footnote-2) for random treatment-effect variance, τ^2^_1_ for different approach, by number of studies, total average sample size, mixture of studies sizes and degree of random effects variances (data generated from random study- and treatment effect: Equation 1 with 5% outcome rate)**

|  |  | Equally sized | | | | | | 25% large studies | | | | | | | | |
| --- | --- | --- | --- | --- | --- | --- | --- | --- | --- | --- | --- | --- | --- | --- | --- | --- |
|  |  | Random-effects Variances (τ^2^_0_, τ^2^_1_)[[2]](#footnote-3) | | | | | | Random-effects Variances (τ^2^_0_, τ^2^_1_) | | | | | | | | |
| (Number of studies, total average sample size) | Methods[[3]](#footnote-4) | (0.05, 0.05) | (0.05, 1) | (0.05, 4) | (1,1) | (1,4) | (4,4) | (0.05, 0.05) | (0.05, 1) | | (0.05, 4) | (1,1) | (1,4) | | (4,4) | |
| (5,500) | Model 1 | 0.158 (0.158, 0.376) | 2.533 (1.357, 3.162) | 7.401 (3.864, 10.733) | 2.384 (1.309, 3.127) | 8.220 (4.345, 11.185) | 7.833 (4.114, 11.147) | 0.158 (0.158, 0.481) | 2.876 (1.687, 3.162) | | 8.639 (4.625, 11.749) | 2.644 (1.496, 3.150) | 8.938 (4.933, 12.006) | | 9.375 (5.615, 11.889) | |
|  | Model 2 | 0.158 (0.158, 0.158) | 3.162 (2.010, 3.162) | 7.627 (3.762, 12.649) | 3.162 (1.820, 3.162) | 8.546 (4.416,12.649) | 8.511 (4.476,12.649) | 0.158 (0.158, 0.158) | 3.162 (3.162, 3.162) | | 10.317 (5.796, 12.649) | 3.162 (3.131, 3.162) | 9.914 (5.455, 12.649) | | 10.342 (5.989, 12.649) | |
|  | Model 3 (PQL) | 0.158 (0.158, 0.158) | 3.162 (3.162, 3.162) | 12.649 (12.649, 12.649) | 3.162 (3.162, 3.162) | 12.649 (12.649, 12.649) | 12.649 (8.566, 12.649) | 0.158 (0.158, 0.158) | 3.162 (3.162, 3.162) | | 12.649 (12.649, 12.649) | 3.162 (3.162, 3.162) | 12.649 (12.649, 12.649) | | 12.649 (12.649, 12.649) | |
|  | Model 3(AGHQ) | 1.042 (0.158, 2.409) | 1.957 (0.924, 3.094) | 5.942 (2.911, 9.125) | 2.332 (1.288, 3.038) | 7.982 (4.024, 10.998) | 8.935 (4.956, 11.935) | 0.158 (0.155, 0.572) | 2.571 (1.719, 3.069) | | 10.406 (7.689, 11.905) | 2.726 (1.923, 3.109) | 10.667 (7.704, 12.200) | | 11.383 (8.186, 12.507) | |
|  | Model 4 (PQL) | 0.158 (0.158, 1.089) | 2.957 (1.324, 3.162) | 7.836 (4.249, 12.277) | 3.028 (1.566, 3.162) | 7.966 (4.043, 12.195) | 7.767 (4.110, 11.965) | 0.158 (0.158, 2.191) | 3.162 (2.194, 4.057) | | 9.287 (4.373, 12.649) | 3.162 (2.085, 3.662) | 9.466 (5.034, 12.649) | | 9.849 (5.090, 12.649) | |
|  | Model 4 (AGHQ) | 0.158 (0.158, 0.158) | 3.086 (1.592, 3.162) | 7.753 (4.165, 11.101) | 3.162 (1.743, 3.162) | 8.419 (4.260, 11.884) | 8.829 (4.622, 12.559) | 0.158 (0.158, 0.158) | 3.162 (2.961, 3.162) | | 10.349 (5.425, 12.649) | 3.162 (2.829, 3.162) | 10.498 (5.167, 12.649) | | 11.144 (6.219, 12.649) | |
| (15, 3000) | Model 1 | 0.158 (0..095, 0.161) | 1.297 (0.639, 2.025) | 4.293 (2.108, 6.584) | 1.357 (0.685, 2.131) | 4.900 (2.516, 7.125) | 4.808 (2.641, 7.272) | 0.152 (0.085, 0.187) | 1.417 (0.742, 2.195) | 4.212 (2.147, 6.589) | | 1.583 (0.827, 2.320) | | 5.811 (3.169, 8.129) | | 6.040 (3.374, 8.546) |
|  | Model 2 | 0.158 (0.158, 0.158) | 1.510 (0.787, 2.256) | 4.441 (2.098, 6.682) | 1.417 (0.686, 2.154) | 4.701 (2.388, 6.959) | 4.731 (2.732, 7.172) | 0.158 (0.158, 0.158) | 1.673 (0.895, 2.422) | 6.159 (3.637, 8.265) | | 1.655 (0.832, 2.365) | | 6.059 (3.374, 8.352) | | 6.132 (3.388, 8.465) |
|  | Model 3 (PQL) | 0.158 (0.158, 0.158) | 3.162 (3.162, 3.162) | 12.649 (12.649, 12.649) | 3.162 (3.162, 3.162) | 8.332 (7.282, 12.649) | 8.006 (7.167, 8.930) | 0.158 (0.158, 0.158) | 3.162 (3.162, 3.162) | 12.649 (12.649, 12.649) | | 3.162 (1.634, 3.162) | | 9.328 (7.621, 12.649) | | 8.367 (7.264, 9.779) |
|  | Model 3(AGHQ) | 0.215 (0.125, 0.759) | 1.056 (0.471, 1.828) | 3.384 (1.600, 5.598) | 1.262 (0.616, 2.114) | 3.688 (1.804, 6.281) | 4.537 (2.266, 6.980) | 0.158 (0.096, 0.407) | 1.502 (0.721, 2.236) | 4.509 (2.174, 7.232) | | 1.497 (0.705, 2.398) | | 4.664 (2.332, 7.553) | | 4.565 (2.178, 7.887) |
|  | Model 4 (PQL) | 0.158 (0.158, 0.391) | 1.141 (0.612, 1.958) | 3.988 (2.070, 6.530) | 1.244 (0.657, 2.059) | 3.702 (1.689, 6.082) | 4.100 (2.048, 6.369) | 0.158 (0.158, 0.235) | 1.440 (0.654, 2.241) | 3.949 (2.025, 6.637) | | 1.433 (0.702, 2.250) | | 4.480 (2.180, 7.094) | | 4.357 (2.121, 7.195) |
|  | Model 4 (AGHQ) | 0.158 (0.158, 0.214) | 1.160 (0.578, 1.904) | 4.086 (1.858, 6.395) | 1.271 (0.608, 1.970) | 4.022 (1.861, 6.204) | 4.299 (2.103, 6.521) | 0.158 (0.158, 0.158) | 1.439 (0.713, 2.274) | 4.474 (2.296, 6.892) | | 1.458 (0.739, 2.212) | | 4.630 (2.493, 7.137) | | 4.707 (2.358, 7.246) |
| (50,9000) | Model 1 | 0.130 (0.076, 0.158) | 1.020 (0.566, 1.480) | 2.783 (1.508, 4.12) | 1.148 (0.607, 1.641) | 4.514 (2.895, 5.831) | 4.816 (3.387, 6.176) | 0.120 (0.066, 0.155) | 0.925 (0.447, 1.464) | | 3.301 (1.761, 4.792) | 1.319 (0.756, 1.771) | 5.335 (3.961, 6.704) | | 6.005 (4.785, 7.197) | |
|  | Model 2 | 0.158 (0.158, 0.158) | 1.497 (0.971, 1.953) | 4.179 (2.591, 5.567) | 1.149 (0.595, 1.649) | 4.348 (2.808, 5.624) | 4.734 (3.237, 6.076) | 0.158 (0.095, 0.158) | 1.417 (0.876, 1.908) | | 6.121 (4.705, 7.238) | 1.365 (0.871, 1.869) | 5.950 (4.571, 7.183) | | 5.907 (4.653, 7.174) | |
|  | Model 3 (PQL) | 0.158 (0.119, 0.192) | 0.980 (0.433, 2.402) | 2.056 (0.975, 3.531) | 0.730 (0.353, 1.201) | 2.605 (1.357, 4) | 2.799 (1.517, 4.323) | 0.158 (0.158, 0.158) | 3.162 (3.162, 3.162) | | 12.642 (7.336, 12.649) | 1.481 (0.543, 3.162) | 7.876 (7.362, 8.511) | | 7.855 (7.431, 8.329) | |
|  | Model 3(AGHQ) | 0.158 (0.087, 0.362) | 0.670 (0.326, 1.196) | 1.886 (0.800, 3.276) | 0.733 (0.351, 1.200) | 2.090 (1.081, 3.681) | 2.306 (1.116, 3.909) | 0.134 (0.067, 0.244) | 0.842 (0.422, 1.392) | | 2.435 (1.198, 4.054) | 0.837 (0.366, 1.404) | 2.473 (1.137, 4.173) | | 2.487 (1.131, 4.239) | |
|  | Model 4 (PQL) | 0.158 (0.099, 0.215) | 0.660 (0.319, 1.117) | 2.149 (0.998, 3.531) | 0.709 (0.352, 1.186) | 2.077 (0.965, 3.583) | 2.512 (1.188, 3.980) | 0.158 (0.082, 0.158) | 0.831 (0.392, 1.356) | | 2.389 (1.213, 3.853) | 0.788 (0.343, 1.298) | 2.495 (1.229, 3.985) | |  | |
|  | Model 4 (AGHQ) | 0.158 (0.105, 0.159) | 0.707 (0.334, 1.140) | 2.339 (1.165, 3.769) | 0.745 (0.362, 1.207) | 2.579 (1.258, 3.983) | 2.828 (1.523, 4.378) | 0.158 (0.090, 0.158) | 0.815 (0.388, 1.340) | | 3.055 (1.425, 4.513) | 0.799 (0.371, 1.318) | 3.010 (1.471, 4.431) | |  | |

1. Percent root mean square error of τ^2^_1_ was calculated for each simulated meta-analysis first, and then summarized across meta-analyses. For each combination of data generation parameters, 1000 meta-analyses were generated. [↑](#footnote-ref-2)
2. τ^2^_0_ is the random study-effect variance and τ^2^_1_, the random treatment-effect variance [↑](#footnote-ref-3)
3. Model 1 (bivariate two-stage); Model 2 (conventional DerSimonian and Laird two-stage); Model 3 (random intercept and random slope one-stage via PQL and AGHQ); Model 4 (stratified intercept one-stage via PQL and AGHQ). [↑](#footnote-ref-4)
