# Supplementary material for: A comparison of analytic approaches for individual patient data meta-analyses with binary outcomes
Source: BMC Med Res Methodol. 2017 Feb 16;17:28. doi: 10.1186/s12874-017-0307-7 (PMC5312561; doi:10.1186/s12874-017-0307-7)
Supplement: Additional file 5: — Percent Coverage (percent convergence rate) for treatment effect, β1 for different approach, by number of studies, total average sample size, mixture of studies sizes and degree of random effects variances - data generated from random study- and treatment effect: Eq. 1 with 5% outcome rate. (DOC 62 kb) [file 12874_2017_307_MOESM5_ESM.doc]

Table S5: Percent Coverage **[[1]](#footnote-2)**(percent convergence rate)**[[2]](#footnote-3)** for treatment effect, β_1_ for different approach, by number of studies, total average sample size, mixture of studies sizes and degree of random effects variances (data generated from random study- and treatment effect: Equation 1 with 5% outcome rate)

|  |  | Equally sized | | | | | | 25% large studies | | | | | | | | |
| --- | --- | --- | --- | --- | --- | --- | --- | --- | --- | --- | --- | --- | --- | --- | --- | --- |
|  |  | Random-effects Variances (τ^2^_0_, τ^2^_1_)[[3]](#footnote-4) | | | | | | Random-effects Variances (τ^2^_0_, τ^2^_1_) | | | | | | | | |
| (Number of studies, total average sample size) | Methods[[4]](#footnote-5) | (0.05, 0.05) | (0.05, 1) | (0.05, 4) | (1,1) | (1,4) | (4,4) | (0.05, 0.05) | (0.05, 1) | | (0.05, 4) | (1,1) | (1,4) | | (4,4) | |
| (5,500) | Model 1 | 96.8 (100) | 92.3 (100) | 88 (100) | 92.6 (100) | 89.4 (100) | 88 (100) | 96.6 (100) | 87.4 (100) | | 83.8 (100) | 87.5 (100) | 86.8 (100) | | 86.5 (100) | |
|  | Model 2 | 97.7 (100) | 93.4 (100) | 89.7 (100) | 89.7 (100) | 86.6 (100) | 83.2 (100) | 97.5 (100) | 83.9 (100) | | 81.4 (100) | 85 (100) | 84 (100) | | 82 (100) | |
|  | Model 3 (PQL) | 94.8 (99.9) | 89.4 (99.7) | 87.2 (99.6) | 89.3 (99.9) | 87 (99) | 85.4 (99.4) | 94.9 (99.6) | 82.7 (99.7) | | 80.9 (99.2) | 84.6 (99.7) | 82.5 (99.3) | | 83.4 (99.1) | |
|  | Model 3(AGHQ) | 95.3 (55.7) | 94.8 (56.7) | 92.6 (61.7) | 96.1 (46.8) | 94.1 (67.6) | 93.8 (65.8) | 79.4 (74) | 73.1 (67.7) | | 71.9 (63.8) | 82.4 (48.9) | 80.5 (53.8) | | 89.7 (51.8) | |
|  | Model 4 (PQL) | 96.1 (93.6) | 92.6 (94.6) | 90.2 (96.3) | 92.7 (71.7) | 91.7 (76.3) | 93.4 (46.9) | 99.1 (34.5) | 95.6 (41.7) | | 95.2 (49.4) | 96.9 (30.5) | 96.5 (39.2) | | 96.3 (27.9) | |
|  | Model 4 (AGHQ) | 94.3 (99.3) | 88.3 (100) | 85.1 (100) | 83.7 (99.7) | 82.3 (100) | 76 (100) | 84.8 (100) | 75.4 (99.7) | | 75 (100) | 71.1 (99.9) | 72.4 (100) | | 68.3 (99.9) | |
| (15, 3000) | Model 1 | 96.2 (100) | 93.6 (100) | 93.3 (100) | 92.7 (100) | 94.4 (100) | 92.5 (100) | 94.5 (100) | 89.5 (100) | 92.3 (100) | | 88 (100) | | 90.5 (100) | | 91 (100) |
|  | Model 2 | 96.2 (100) | 93.6 (100) | 92.3 (100) | 88.3 (100) | 92.1 (100) | 88.3 (100) | 94.9 (100) | 88.5 (100) | 91 (100) | | 86.6 (100) | | 90.3 (100) | | 86.6 (100) |
|  | Model 3 (PQL) | 92.5 (96.8) | 89.8 (78.3) | 94.8 (83.6) | 87.7 (94.1) | 94.3 (95.2) | 90.9 (98.4) | 91.4 (94.7) | 86.9 (84.4) | 92.4 (88) | | 81.6 (95.3) | | 86.7 (96.3) | | 86.9 (97.6) |
|  | Model 3(AGHQ) | 97.6 (61.2) | 93.7 (83.9) | 93.3 (86.3) | 93.7 (94.1) | 93.6 (99.8) | 93.2 (99.7) | 92.7 (58) | 86.5 (75.6) | 87.5 (82.8) | | 92.6 (84.7) | | 91.5 (97.3) | | 92.9 (98.5) |
|  | Model 4 (PQL) | 94.4 (99.7) | 93.5 (99.7) | 93.7 (99.7) | 92.3 (75.2) | 94.5 (81.9) | 97.7 (26.8) | 96.5 (48.6) | 94.9 (56.8) | 95.4 (67.5) | | 97.2 (20.1) | | 97 (33.8) | | 99.2 (6.5) |
|  | Model 4 (AGHQ) | 93.9 (99.8) | 92 (100) | 92.2 (100) | 87.8 (100) | 90.9 (100) | 88 (100) | 86.2 (99.8) | 85.8 (100) | 89.6 (100) | | 75.4 (100) | | 86 (100) | | 77.6 (100) |
| (50,9000) | Model 1 | 96.2 (100) | 94.7 (100) | 94.2 (100) | 94.4 (100) | 93 (100) | 92.8 (100) | 94.6 (100) | 93.5 (100) | | 93.9 (100) | 88.7 (100) | 90.6 (100) | | 89.2 (100) | |
|  | Model 2 | 96.8 (100) | 95.2 (100) | 93.6 (100) | 78 (100) | 87.4 (100) | 76.5 (100) | 94.5 (100) | 93.3 (100) | | 93.6 (100) | 83.6 (100) | 86.1 (100) | | 77.2 (100) | |
|  | Model 3 (PQL) | 98.6 (26.6) | 99.1 (24) | 98 (22.3) | 97.7 (37.7) | 95.4 (63.8) | 92.8 (96.1) | 91.4 (94.7) | 91.8 (89.1) | | 95.4 (59.9) | 95.7 (23.4) | 96.4 (27.3) | | 93.6 (90.6) | |
|  | Model 3(AGHQ) | 96.6 (93.7) | 95.4 (95.7) | 93.3 (96.9) | 94.1 (99.9) | 92.7 (100) | 94.7 (100) | 97.2 (65.6) | 93.4 (90.1) | | 91.3 (96) | 93.4 (100) | 93.9 (100) | | 94.9 (100) | |
|  | Model 4 (PQL) | 96.4 (65.1) | 95.6 (88) | 93.7 (97.8) | 96.6 (29.9) | 94.7 (49.6) | 100 (0.90) | 99.9 (2.8) | 99.8 (6.8) | | 98.9 (16.3) | 99.9 (0.3) | 99.9 (0.8) | |  | |
|  | Model 4 (AGHQ) | 94.2 (100) | 94.3 (100) | 92.6 (100) | 82.6 (100) | 88.4 (100) | 80.4 (100) | 51.9 (100) | 81.8 (100) | | 91.9 (100) | 63.9 (100) | 83.3 (100) | |  | |

1. Percent coverage of β_1_ was calculated for each simulated meta-analysis first, and then summarized across meta-analyses. For each combination of data generation parameters, 1000 meta-analyses were generated. [↑](#footnote-ref-2)
2. Numerical convergence was only reported for PQL and AGHQ via GLIMMIX procedure in SAS. [↑](#footnote-ref-3)
3. τ^2^_0_ is the random study-effect variance and τ^2^_1_, the random treatment-effect variance [↑](#footnote-ref-4)
4. Model 1 (bivariate two-stage); Model 2 (conventional DerSimonian and Laird two-stage); Model 3 (random intercept and random slope one-stage via PQL and AGHQ); Model 4 (stratified intercept one-stage via PQL and AGHQ). [↑](#footnote-ref-5)
