# Supplementary material for: A comparison of analytic approaches for individual patient data meta-analyses with binary outcomes
Source: BMC Med Res Methodol. 2017 Feb 16;17:28. doi: 10.1186/s12874-017-0307-7 (PMC5312561; doi:10.1186/s12874-017-0307-7)
Supplement: Additional file 6: — Percent Coverage for random treatment-effect variance, τ2 1 for different approach, by number of studies, total average sample size, mixture of studies sizes and degree of random effects variances - data generated from random study- and treatment effect: Eq. 1 with 5% outcome rate. (DOC 63 kb) [file 12874_2017_307_MOESM6_ESM.doc]

Table S6: Percent Coverage**[[1]](#footnote-2)** for random treatment-effect variance, τ^2^_1_ for different approach, by number of studies, total average sample size, mixture of studies sizes and degree of random effects variances (data generated from random study- and treatment effect: Equation 1 with 5% outcome rate)

|  |  | Equally sized | | | | | | 25% large studies | | | | | | | | |
| --- | --- | --- | --- | --- | --- | --- | --- | --- | --- | --- | --- | --- | --- | --- | --- | --- |
|  |  | Random-effects Variances (τ^2^_0_, τ^2^_1_)[[2]](#footnote-3) | | | | | | Random-effects Variances (τ^2^_0_, τ^2^_1_) | | | | | | | | |
| (Number of studies, total average sample size) | Methods[[3]](#footnote-4) | (0.05, 0.05) | (0.05, 1) | (0.05, 4) | (1,1) | (1,4) | (4,4) | (0.05, 0.05) | (0.05, 1) | | (0.05, 4) | (1,1) | (1,4) | | (4,4) | |
| (5,500) | Model 1 | NA | NA | NA | NA | NA | NA | NA | NA | | NA | NA | NA | | NA | |
|  | Model 2 | NA | NA | NA | NA | NA | NA | NA | NA | | NA | NA | NA | | NA | |
|  | Model 3 (PQL) | 100 :9.3 | 95.2 :6 | 57.1 :2.8 | 98.3 :5.9 | 35.9 :5.6 | 21.8 :8 | 100 :8.2 | 98.1 :5.2 | | 40.9 :1.8 | 93.8 :4.5 | 41.6 :3.2 | | 34.9 :7.1 | |
|  | Model 3(AGHQ) | 37.2 :5.5 | 76 :16.5 | 84.9 :32.9 | 90.6 :29.9 | 80.6 :46.9 | 82.8 :49.9 | 27.9 :3.1 | 58.8 :7.7 | | 62.8 :12.9 | 68.2 :9.3 | 67.3 :17.5 | | 77.2 :28.5 | |
|  | Model 4 (PQL) | 100 :40.5 | 99.2 :71.6 | 86.5 :78.5 | 96.8 :52.1 | 84.3 :60.1 | 84.3 :37.7 | 100 :16.2 | 99.7 :28.3 | | 93.1 :40.7 | 96.7 :20.8 | 89.5 :31.6 | | 79.4 :20.5 | |
|  | Model 4 (AGHQ) | 60.7 :25.2 | 77.6 :57.8 | 70.2 :65.8 | 76.7 :52.8 | 66.4 :59 | 68 :55.2 | 52.6 :12.4 | 74.4 :35 | | 74.8 :53.1 | 80.6 :35.8 | 75.4 :51.5 | | 68.9 :46.2 | |
| (15, 3000) | Model 1 | NA | NA | NA | NA | NA | NA | NA | NA | NA | | NA | | NA | | NA |
|  | Model 2 | NA | NA | NA | NA | NA | NA | NA | NA | NA | | NA | | NA | | NA |
|  | Model 3 (PQL) | 97.1 :3.5 | 100 :2.7 | 26.2 :5.6 | 98.3 :222.9 | 0 :0 | 0.3 :0.3 | 96.2 :2.6 | 97.1 :7.4 | 25.2 :4.2 | | 94.2 :24.7 | | 0.4 :0.2 | | 0 :0 |
|  | Model 3(AGHQ) | 93.5 :39.7 | 89.5 :69.2 | 82.6 :68.6 | 86.1 :80.6 | 84.9 :84.7 | 85.9 :85.6 | 91.3 :22.9 | 84.8 :49.4 | 72.8 :53.2 | | 86.5 :69.9 | | 81.7 :79.2 | | 83 :81.4 |
|  | Model 4 (PQL) | 100 :57.2 | 89.9 :88.9 | 88.9 :88.6 | 87 :65 | 87.2 :71.4 | 87.7 :23.5 | 100 :25.3 | 84.8 :46.4 | 85.6 :57.6 | | 91.8 :17.8 | | 87.9 :29.7 | | 89.2 :5.8 |
|  | Model 4 (AGHQ) | 83.5 :48 | 84.1 :83.4 | 80.3 :80.3 | 81.2 :80.4 | 79.2 :79.2 | 76 :75.8 | 86.1 :36.5 | 79.6 :73.9 | 76 :75.8 | | 82.5 :72.1 | | 75.4:75.1 | | 76.1:73.9 |
| (50,9000) | Model 1 | NA | NA | NA | NA | NA | NA | NA | NA | | NA | NA | NA | | NA | |
|  | Model 2 | NA | NA | NA | NA | NA | NA | NA | NA | | NA | NA | NA | | NA | |
|  | Model 3 (PQL) | 100 :21.1 | 88.3 :21.2 | 89.4 :19.9 | 91 :34.3 | 79.3 :50.6 | 75.2 :72.3 | 88.9 :0.8 | 97.1 :6.8 | | 11.1 :3.4 | 56.3 :2.7 | 0 :0 | | 0 :0 | |
|  | Model 3(AGHQ) | 93.3 :80 | 87.3 :86.5 | 86.9 :84.9 | 89.8 :89.8 | 91.3 :91.3 | 92.1 :92.1 | 98.7 :54 | 89.7 :76.8 | | 83.1 :78.6 | 88.5 :88.4 | 91.8 :91.8 | | 92.1 :92.1 | |
|  | Model 4 (PQL) | 99.4 :34.4 | 90.8 :79.9 | 92.3 :90.3 | 87 :26 | 91.3 :45.3 | 77.8 :0.7 | 100 :1.2 | 91.2 :6.2 | | 87.1 :14.2 | 100 :0.3 | 100 :0.8 | | 100 :0.8 | |
|  | Model 4 (AGHQ) | 94.5 :91.6 | 87.6 :87.6 | 85 | 84.4 :84.4 | 80.8 :80.8 | 76.7 :76.7 | 99.7 :29.2 | 87.2 :82.7 | | 79.3 :79.3 | 90 :80.2 | 79.8 :79.8 | | 79.8 :79.8 | |

1. Percent coverage of τ^2^_1_ was calculated assuming normality for each simulated meta-analysis first, and then summarized across meta-analyses. For each combination of data generation parameters, 1000 meta-analyses were generated. Coverage was reported as a ratio of the subset of cases that excluded meta-analyses where no standard error was estimated, to cases that included these meta-analyses as non-coverage. [↑](#footnote-ref-2)
2. τ^2^_0_ is the random study-effect variance and τ^2^_1_, the random treatment-effect variance [↑](#footnote-ref-3)
3. Model 1 (bivariate two-stage); Model 2 (conventional DerSimonian and Laird two-stage); Model 3 (random intercept and random slope one-stage via PQL and AGHQ); Model 4 (stratified intercept one-stage via PQL and AGHQ). [↑](#footnote-ref-4)
